# Magnetically responsive electrospun fibers as programmable bioactive scaffolds: From formulation design to magnetically triggered therapy and regeneration

**DOI:** 10.1016/j.bioactmat.2026.08.014

**Published:** 2026-08-12

**Authors:** Ashish Trital, Burhan Ates, Ryan J Gilbert

**Affiliations:** a Linda and Bipin Doshi Department of Chemical and Biochemical Engineering, Missouri University of Science and Technology, Rolla, MO, 65409, United States; b Department of Chemistry, Faculty of Arts and Sciences, Inonu University, Malatya, 44280, Turkey

**Keywords:** Electrospinning, Magnetic particles, Alternating magnetic field, Hyperthermia, Controlled release, Localized therapy, Biomedical application

## Abstract

Magnetically responsive electrospun fibers (MREFs) represent an emerging class of active soft materials designed to control where, when, and how fast a therapeutic agent or biophysical cue is delivered. Unlike conventional electrospun fibers, whose release and mechanical behavior are fixed at fabrication, MREFs can be manipulated after implantation, using an applied magnetic field to generate localized heating, mechanical deformation, structural reorientation, magnetic guidance, or triggers thermoresponsive release. This review compares MREF compositions, fabrication strategies, and magnetothermal, magneto-mechanical, and magnetic-guidance mechanisms across applications in cancer therapy, tissue regeneration, wound healing, and theranostics and critically evaluates key translational barriers, safety, and regulatory requirements. Current evidence suggests that localized and surface-accessible treatments are the most realistic near-term clinical applications.

## Highlights

• MREFs integrate fibrous biomimetic scaffolds with remote magnetic responsiveness.
• Material composition and architecture govern MREF function.
• AMF heating enables hyperthermia and on-demand therapeutic release.
• Magnetic guidance and actuation support tissue organization and regeneration.
• Clinical translation requires scalable production and safe delivery of magnetic fields.

## Graphical abstract

Magnetically responsive electrospun fibers (MREFs) for biomedical applications. MREFs are electrospun fiber scaffolds integrated with magnetic fillers and therapeutic payloads. Externally applied magnetic fields enable remote activation, controlled drug release, localized stimulation, and multifunctional biomedical use.

**Figure:**
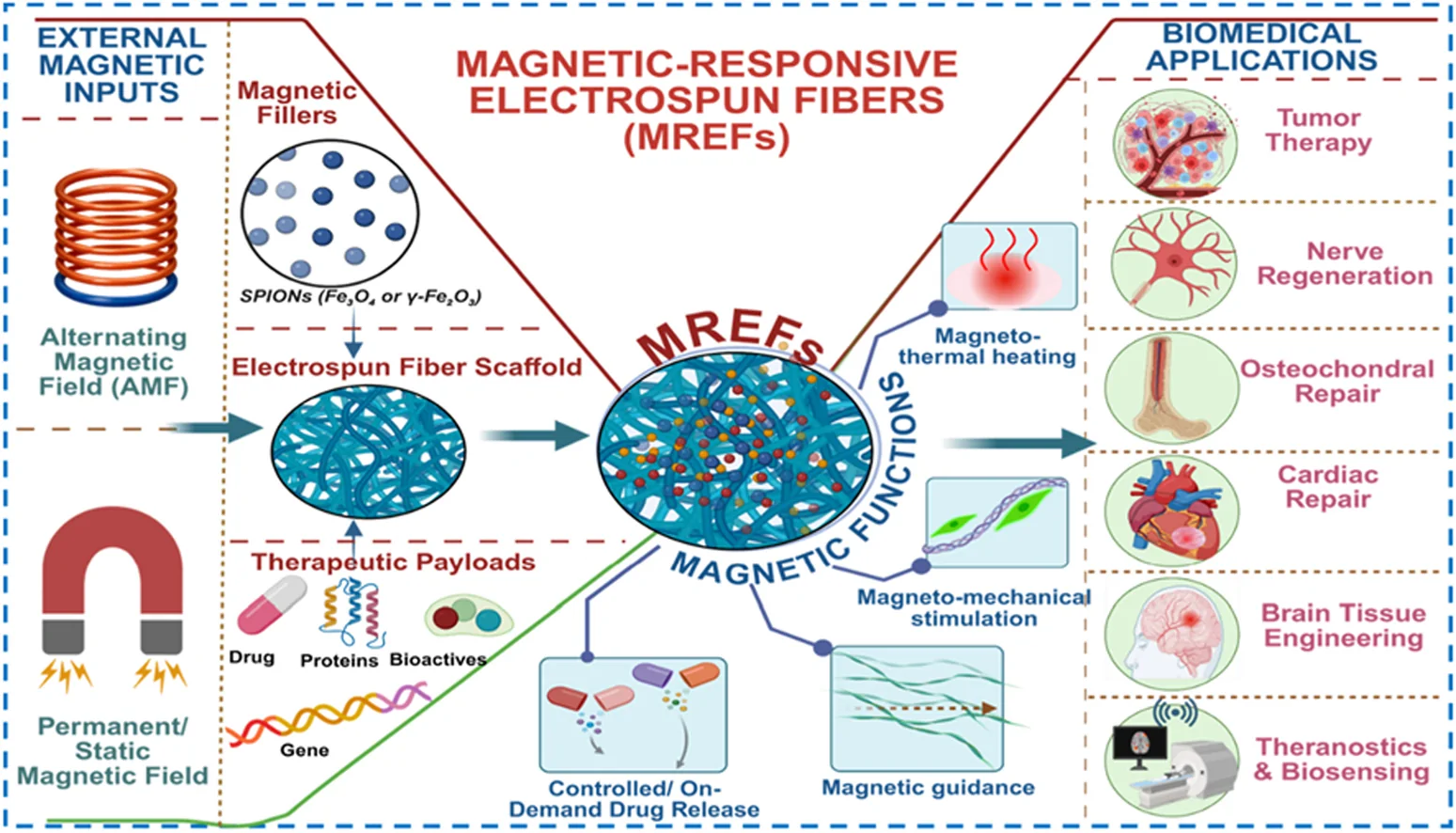

## 1 Introduction

Biomaterial designs that respond to defined physical and chemical stimuli are increasingly important for the development of advanced clinical materials [1]. Among these, stimuli-responsive platforms have attracted growing attention because they can provide controlled, site-specific, and on-demand therapeutic functions. Magnetically responsive biomaterials are particularly attractive because they enable non-invasive actuation, allowing biomaterial systems to be activated in deep anatomical locations that are difficult to reach using optical or electrochemical triggers [2,3]. Electrospun fibers are known for their ability to mimic the fibrillar architecture of the native extracellular matrix [4]. Incorporating magnetic fillers into these fibers produces magnetically responsive electrospun fibers (MREFs), which provide a highly versatile and biological relevant platform with broad applicability in tissue engineering, drug delivery, regenerative medicine, magnetic hyperthermia, and other biomedical applications [5,6]**.**

Electrospinning is one of the most widely used fabrication methods for developing fibrous scaffolds, as it generates micro-to-nano scale fibers with high porosity and surface area that resemble the architecture of the native extracellular matrix [4]. These characteristics have enabled electrospun fibers to be used in drug delivery, tissue engineering, wound healing, implant coating and biosensing [7,8]. Recent publications demonstrate the ability of fibers to respond to external stimuli like pH, temperature, light, electrical fields, and magnetic fields [[9], [10], [11]]. Redox-responsive fibers provide an additional release mechanism by using disulfide or thioether bonds that cleave under the high glutathione levels found inside cells and in tumor microenvironments. However, direct integration of redox responsiveness with magnetic electrospun fibers remains limited and should be presented as an emerging multi-stimulus design strategy rather than an established MREF class [12]. Among these approaches, magnetically responsive fiber designs are especially attractive for major biomedical applications because they combine biomimetic scaffold architecture with noninvasive remote control, and multifunctional therapeutic capability [5,13].

Iron oxide nanoparticles (IONP) especially magnetite (Fe_3_O_4_) and maghemite (γ-Fe_2_O_3_) are the dominant magnetic fillers incorporated into electrospun fibers, because their magnetic properties, surface chemistry and biomedical precedents are better established than those of most alternative ferrites or metallic fillers [14,15]. When applying an alternating magnetic field (AMF) at frequencies of 100 kHz to 1 MHz, IONP dissipate magnetic energy and generate localized heat through relaxation losses, with heating efficiency depending on particle size, field parameters and local viscosity [16,17]. Magnetic hyperthermia forms the foundation for several European regulatory clinical applications translated through Nano Therm ® therapy (MagForce AG) for glioblastoma and provides an example of the translational potential of an IONP-based platform [[18], [19], [20]]. Early magnetic responsive biomaterial research focused on magnetic nanoparticles for imaging, targeting, and hyperthermia, whereas later work integrated these nanoparticles into hydrogels, polymer scaffolds, and fibrous matrices to provide spatial control, mechanical support, and localized therapeutic function [21,22]. Immobilization within fibers may reduce immediate particle dispersion compared with freely injected suspensions. Magnetic particles embedded within a polymer matrix may also behave differently from free suspensions because polymer-particle interactions, aggregation and restricted rotational motion alter heating, force generation and release behavior [5,16,17]. Ganguly et al. demonstrated magnetic-field-dependent diffusion control using amine-functionalized maghemite incorporated into a hydrogel network for magnetic-field-controlled, diffusion-driven release [23] while broader theranostic studies show how magnetic fillers can combine imaging and therapeutic functions, with performance determined by particle architecture and magnetic organization [24].

The volume of primary literature on MREFs platforms has grown exponentially over the past two decades. Nevertheless, no MREF-based product is in clinical use, and the gap between prolific laboratory-scale publications and clinical translation remains substantial. Existing reviews mainly address magnetic nanofiber fabrication [5], cancer hyperthermia [22], theranostics [25], or tissue engineering [26]. In contrast, this review integrates magnetothermal, magneto-mechanical, and magnetic-guidance mechanisms within a single design framework. It compares fabrication routes and magnetic fillers using practical selection criteria, and critically evaluates biosafety, field-delivery, dose, manufacturing, and regulatory barriers to clinical translation. Table 1 summarizes this distinction, while Sections 6, 7 provide the review's principal contribution through a focused analysis of long-term safety and translational feasibility.

**Table 1:** Positioning of the present review relative to recent reviews on related topics.

Review scope	Fabrication strategy	Magnetic filler comparison	Magneto-thermal	Magneto-mechanical	Magnetic guidance	Biosafety/biodistribution	Human-scale AMF delivery	Manufacturing & regulatory	Year/References
Magneto-responsive scaffolds (general)	○	◐	◐	●	◐	◐	○	**○**	2018 [21]
Fabrication	◐	◐	●	○	◐	○	○	**○**	2021 [5]
Theranostic	○	◐	●	○	○	○	○	**○**	2021 [25]
Cancer hyperthermia	◐	◐	●	○	○	◐	◐	○	2023 [22]
Tissue engineering	○	◐	◐	●	◐	○	○	○	2024 [26]
MREFs	●	●	●	●	●	●	●	●	Current review

● = major focus; ◐ = moderate; **○** = limited. *Symbols indicate the relative emphasis of each review based on the breadth and depth of discussion rather than quantitative scoring.*

## 2 Materials used in magnetic-responsive electrospun fibers

### 2.1 Polymeric matrices

Polymer selection is a crucial step in designing MREFs as it governs magnetic-particle dispersion, polymer-particle interaction, degradation profile, water uptake, and drug release kinetics. The polymer matrices provide a structural scaffold that mimics extracellular architecture, acts as a carrier for therapeutic payloads and creates the chemical environment for magnetic filler dispersion. Natural, synthetic, and hybrid polymers have been used as electrospinning matrices for magnetic composites [5,27] which are summarized in Table 2. The most suitable polymer is therefore not the one that is electrospun most easily, but the one that supports the intended magnetic function while minimizing formulation complexity and long-term safety concerns.

**Table 2:** Polymer selection for MREFs design.

Polymer/matrix	Advantages in MREFs	Limitation	Application	Refs
PCL	Hydrophobic, slow degradation, easy to electrospun	Slow degradation; weak cell adhesion unless modified	Tumor therapy, nerve, bone repair, wound dressing, cardiac patch	[[28], [29], [30]]
PLGA	Tunable degradation, drug-release control, core–shell drug delivery	Acidic degradation products; possible burst release	Local chemotherapy, post-surgical implants	[[31], [32], [33], [34]]
PLA	Provides mechanical support and stable fiber structure	Brittle; hydrophobic	Bone repair, tissue scaffolds	[35]
PLLA	Useful for structural MREF scaffolds, semi-crystalline, mechanically strong	Slow degradation; limited wettability	Bone tissue engineering, osteochondral repair, nerve repair	[36,37]
PGA	High crystallinity, rapid scaffold resorption	Brittle; fast degradation may reduce stability	Short-term regenerative scaffolds	[33,38]
PBS	Biodegradable, flexible, thermally processable, and readily electrospun	Hydrophobic; limited cell adhesion without surface modification; batch variability	Magnetoresponsive drug delivery, wound dressings, and soft-tissue scaffolds	[[39], [40], [41]]
PHBV	Hydrophobic, mechanically stable, biodegradable matrix, magnetic hybrid designs	Less common in direct MREF studies than PCL/PLGA/PLLA	Wound dressing, tissue regeneration	[42]
PU	Elastic, flexible, mechanically durable or soft-tissue MREFs	Biodegradation depends on formulation	Wound dressing, cardiac patch, soft tissue	[[43], [44], [45]]
PVA	Water-soluble, biocompatible, highly hydrophilic, swelling-controlled release.	Requires crosslinking for water stability	Wound healing, topical drug delivery	[[46], [47], [48]]
PEO	Water soluble, spinning aid for chitosan, alginate, or protein polymers	Dissolves rapidly in aqueous media	Blend component, wound dressing	[49,50]
PEG	Tunes drug diffusion, swelling, surface hydration, protein-resistance, improves wettability	Poor fiber stability alone	Drug release, antifouling surfaces, blends	[51,52]
PNIPAAm/PNIPAm	LCST near 32 °C; temperature-responsive swelling	Weak mechanical stability; difficult to electrospin alone	Smart drug delivery, cancer hyperthermia	[53,54]
PVDF	Piezoelectric, chemically stable, mechanically strong	Poor biodegradability	Cardiac repair, nerve stimulation, biosensing	[55,56]
PAN	Thermally stable, easy to electrospin	Not biodegradable	Biosensing, conductive/magnetic composite fibers	[57,58]
PMMA	Transparent, stable, mechanically rigid, suitable for incorporating therapeutic agents or magnetic nanoparticles	Non-biodegradable, limited long-term tissue integration	Model drug-delivery systems, implant coatings, biosensors, proof-of-concept magnetic composite fibers	[59,60]
Polypyrrole (PPy)	Adds electrical conductivity to magnetic nanofibers	Possible stiffness increase; conductivity and cytocompatibility depend on coating quality	Peripheral nerve repair, cardiac stimulation, biosensing	[61]
Polyaniline (PANI)	Conductive, redox-active, magneto-electrical composite potential	Biocompatibility and processability require careful formulation	Biosensing, neural/cardiac scaffolds, magneto-electrical fibers	[62]
PEDOT/PEDOT:PSS	High conductivity, flexible, widely used in bioelectronics	Direct MREF examples are fewer than PPy/PVDF systems	Nerve conduits, cardiac patches, bioelectronic scaffolds	[63]
Gelatin	Cell-adhesive, biodegradable and tissue integration	Requires crosslinking; poor wet stability	Wound healing, bone, nerve, cartilage	[[64], [65], [66]]
Chitosan	Cationic, biodegradable, antimicrobial, hemostatic	Poor electrospinnability alone; usually blended	Wound healing, antimicrobial dressing	[66,67]
Collagen	Highly bioactive, cell-adhesive, supports tissue regeneration and cell signaling	Expensive; weak mechanical stability; crosslinking needed	Skin, nerve, bone, cardiac tissue	[65,68]
Silk fibroin	Provides mechanical strength and long-term tissue support, slow degradation	Processing affects structure and reproducibility	Bone, cartilage, wound repair	[69,70]
Alginate	Hydrophilic, gel-forming, useful for hydrophilic payload release	Weak mechanics; difficult to electrospin alone	Wound healing, soft-tissue scaffolds	[49]
Cellulose	Hydrophilic, mechanically reinforcing, bioactive composite	Needs blending/crosslinking for electrospinning	Wound healing, antimicrobial dressing	[43,48]
Hyaluronic acid	Hydrophilic, supports cell migration, hydration, and tissue repair	Poor mechanical strength; difficult electrospinning alone	Wound healing, cartilage repair, soft tissue	[71,72]
Pectin	Bioadhesive, hydrophilic, biodegradable	Poor electrospinnability alone; usually requires PVA/PEO blending	Diabetic wound healing, topical dressings	[73]
Cellulose acetate phthalate (CAP)	pH-sensitive, film/fiber-forming, drug-compatible, hyperthermia	Hydrophobicity and pH-dependent behavior must match target site	Tumor therapy, local drug delivery	[74,75]

Natural polymers such as collagen, gelatin, chitosan, silk fibroin, hyaluronic acid derivatives, and fibrin, provide an intrinsic bioactive signal [27]. Their biological advantage is offset by variable composition, limited wet stability leading to batch-to-batch variability and less predictable degradation. Chitosan illustrates this trade-off because its high positive charge density restricts jet elongation and often requires blending with synthetic carriers [[76], [77], [78]]. Therefore, natural polymers are most useful when biological signaling is a primary design objective, whereas natural-synthetic polymer blends offer advantages when both bioactivity and process stability are required, despite the additional formulation complexity.

Among synthetic biodegradable polymers, polycaprolactone (PCL) is well established for MREF fabrication because of its excellent electrospinnability, biocompatibility, mechanical flexibility and slow hydrolytic degradation, which is often reported over a multi-year time scale [79,80]. PCL also has regulatory and clinical precedent in medical products in FDA-approved implantable and injectable products [81]. The major limitation is hydrophobicity, which reduces cell adhesion and proliferation. However, this limitation is often addressed by plasma treatment, hydrolysis and ammonolysis or blending with gelatin or collagen and coaxial spinning with hydrophilic shells [[82], [83], [84], [85], [86]]. Poly (lactic-co-glycolic acid) (PLGA) offers a tunable degradation kinetics from weeks to months by varying the lactide: glycolide ratio, providing control over both scaffold design and release kinetics [34]. A 50:50 composition degrades fastest, while lactide-rich composition degrades more slowly. However, the acid byproduct from PLGA hydrolysis must be considered in pH sensitive release and for bone-regeneration applications [87,88] because local acidification inhibits osteoblast mineralization and reduces alkaline phosphatase activity [89,90]. Polyurethane (PU) is preferable when repeated deformation is required for magneto-mechanical actuation, whereas poly(vinyl alcohol) (PVA) is valuable for aqueous processing but requires stabilization against dissolution [91,92]. These matrices should therefore be selected according to residence time and actuation mode rather than familiarity alone.

Poly(butylene succinate) (PBS) deserves specific attention as a matrix for magnetic scaffolds. PBS is an aliphatic polyester which is more ductile compared to PLA/PLLA, degrades faster than PCL, and has a less acidic degradation profile than PLGA while providing a better balance between scaffold persistence and particle clearance. PBS is readily electrospun and can accommodate relatively high inorganic filler loadings while maintaining jet stability. Scirè et al. recently develop PBS nanofibrous scaffolds containing ciprofloxacin and iron oxide nanoparticles that combined superparamagnetic behavior, imaging visibility and NIR-triggered photothermal drug release [41]. Broader PBS studies have demonstrated controlled antibacterial delivery and wound treatment potential [39] while PBS based copolyester scaffolds have provided porous and elastic architectures for soft-tissue engineering [40]. However, evidence for reproducible magnetic loading, long-term degradation and *in vivo* particle fate remains limited, so PBS should be viewed as a promising matrix rather than an established replacement for PCL or PLGA.

Thermoresponsive polymers provide additional functionality in MREF design that enables temperature-regulated swelling, diffusion, and drug release. Poly(N-isopropylacrylamide) (PNIPAAm) is most studied, with a lower critical solution temperature (LCST) near 32 °C and co-polymerization with acrylamide or acrylic acid shifts the LCST into the 32 °C-42 ^°^C range [93]. Above the LCST, the network undergoes a hydrophilic-to-hydrophobic transition, changing from a swollen drug-permeable state to a contracted barrier. However, direct aqueous electrospinning of PNIPAAm is challenging because its low glass-transition temperature and hydrophilicity limit jet stability and promote fiber fusion after deposition [94,95]. This limitation is addressed by blending with polymers PVA, PCL, or PU, that stabilize fiber morphology while retaining thermoresponsiveness, although dilution of the responsive phase may reduce the magnitude and sharpness of the LCST transition. [94,96]. Lin et al. demonstrated temperature-controlled nifedipine release from PNIPAAm/PU nanofibers [94] while Hu et al. showed reversible wettability changes across the LCST in PNIPAAm/ethyl cellulose nanofibers that switched from hydrophilic to hydrophobic to support cell growth [95]. Gonçalves et al. shift of LCST in p(NIPAAm-co-HMAAm) to approximately 40 °C and achieved temperature-enhanced doxorubicin release under acidic conditions [97]. These studies confirm the feasibility of thermoresponsive fibers, but they do not establish magnetic control unless the transition is activated directly by magnetic heating. Polymer choice therefore matches mechanical and biological function. Elastomeric PU is better suited to magneto-mechanical deformation, slowly degrading PCL provide extended residence times for implantable devices and collagen, or silk fibroin provide stronger biological signaling for neural or musculoskeletal applications [5,21].

### 2.2 Magnetic fillers

The magnetic filler terminology in this field is used inconsistently, which impedes comparison between studies. Throughout this review we adopt the following convention. *Magnetic filler* refers to any magnetically active solid incorporated into a fiber. *Magnetic nanoparticle (MNP)* denotes a filler with all dimensions below 100 nm, whereas *iron oxide nanoparticle (IONP)* specifically refers to magnetite or maghemite nanoparticles without implying a particular magnetic state. *SPION* is reserved for IONP that exhibit superparamagnetism at body temperature, typically below approximately 20–25 nm for magnetite. Larger blocked or multidomain iron oxide particles are termed ferrimagnetic iron oxide particles because their magnetic and heating behaviors differ. When a study does not adequately characterize magnetic state, we use the neutral term *iron oxide particle*. Using these definitions, this review identifies the research priorities required to advance MREFs from laboratory studies toward clinically relevant technologies.

Magnetic fillers are the functional core of MREFs as they determine the actuation mode, heating efficiency, imaging capability and translational risk. Their selection should therefore be based on magnetic state, morphology and biological fate rather than magnetization alone. Magnetic fillers in Table 3 are classified according to the physical properties governing their response, while Table 4 compares their relative suitability for heating, imaging and mechanical actuation. This distinction is important because magnetite and maghemite particles of similar composition can behave differently when they are superparamagnetic, blocked, multidomain or anisotropic.

**Table 3:** Magnetic filler classes for MREF design.

Class	Representative fillers	Physical feature	Advantages	Limitations	Refs
Superparamagnetic iron oxides	Spherical Fe_3_O_4_ or γ-Fe_2_O_3_, typically < ∼20–25 nm	Negligible remanence and coercivity at 37 °C	MRI, magnetothermal release, implantable systems	Lower magnetic moment; heating may decrease after fiber immobilization	[14,15,98,99]
Blocked/multidomain iron oxides	Larger Fe_3_O_4_ or γ-Fe_2_O_3_ particles, multidomain above ∼80–100 nm	Remanence, coercivity and hysteresis heating	Magnetic guidance, mechanical actuation, hyperthermia	Aggregation, jet instability and limited clinical precedent	[22,[100], [101], [102]]
Anisotropic iron oxides	Nanocubes, octopods, nanorods, nanodiscs	Shape-dependent magnetic anisotropy	Enhanced heating and directional actuation	Complex synthesis and difficult alignment within fibers	[29,103]
Doped ferrites	CoFe_2_O_4_, MnFe_2_O_4_, ZnFe_2_O_4_, NiFe_2_O_4_	Tunable anisotropy and magnetostriction	Magneto-mechanical actuation, MRI and hyperthermia	Possible Co, Ni or Mn ion toxicity	[[104], [105], [106], [107], [108], [109], [110]]
Iron-doped bioactive ceramics	Fe-doped calcium phosphate or β-tricalcium phosphate	Combines magnetism with osteoconductivity	Bone regeneration and imageable scaffolds	Weak heating and magnetic actuation	[[111], [112], [113]]
Protein-caged iron oxides	Ferritin, magnetoferritin	Iron oxide enclosed within a protein cage	Biologically compatible magnetic delivery	Low magnetic loading and limited heating	[114,115]
Magnetic metal–organic frameworks	MIL-88(Fe), MIL-53(Fe)	Porous magnetic structure with high drug loading	Drug delivery and theranostic systems	Uncertain stability, degradation and metal release	[116,117]
Magnetic alloys	FeCo, FePt, Ni-based alloys	Very high magnetization and anisotropy	Strong heating, sensing and magnetic actuation	Oxidation, persistence and metal toxicity	[[118], [119], [120]]

Note: Size thresholds are approximate and depend on composition, anisotropy, temperature, aggregation and measurement conditions.

**Table 4:** Comparative performance of magnetic filler classes for MREF design.

Filler class	Magnetic actuation potential	Heating after immobilization	MRI detectability	Relative cytocompatibility	Translational maturity	Preferred MREF role
Iron oxide formulations Fe_3_O_4_/γ-Fe_2_O_3_	++	++	++++	++++	++++ the only class with approved human precedent (ferumoxytol; NanoTherm®)	MRI tracking, AMF-triggered release and mild heating
Blocked or multidomain iron oxide	++++	+++	+++	+++	+++ Preclinical	Magnetic guidance, mechanical actuation and hysteretic heating
Anisotropic iron oxide	+++	++++	+++	+++	+++ Preclinical; scale-up remains difficult	Hyperthermia-focused systems
CoFe_2_O_4_	++++	++++	++	+	+ Exploratory; major ion-release concern	Magnetostrictive and magnetoelectric scaffolds
MnFe_2_O_4_	+++	+++	++++	++	++ Preclinical	Combined imaging and heating
ZnFe_2_O_4_	++	++	++	+++	+++ Preclinical	Lower-toxicity ferrite designs
NiFe_2_O_4_ or Ni alloys	++++	+++	++	+	+ Exploratory; limited implant suitability	Non-implantable or proof-of-concept actuation
Fe-doped calcium phosphate	+	+	+++	++++	++++ Preclinical	MRI-visible bone scaffolds
Ferritin or magnetoferritin	+	+	++	++++	+++ Exploratory	Biodegradable or low-persistence systems
Fe- MOFs	++	++	++	++ Uncertain	+ Exploratory	High payload theranostic fibers
FeCo/FePt	++++	++++	++	+/++	+ Exploratory; persistence and toxicity concerns	Sensing and *ex vivo* actuation

Note: Ratings are relative within this table, (+(low) to ++++ (very high)) and are intended for preliminary material selection rather than as substitutes for matrix-specific experimental measurements. Performance depends on particle size, morphology, coating, loading, aggregation, matrix immobilization and magnetic-field conditions [15,16,98,99,121].

Superparamagnetic iron oxides (SPION) remain the dominant fillers in MREFs because they combine MRI visibility, established surface chemistry and negligible remanence after field removal [14,15,20]. Their principal limitation is that immobilization within a polymer suppresses Brownian rotation, so heating values obtained in suspension may overestimate performance in the final fiber. Larger blocked or multidomain particles are advantageous when magnetic force, rather than magnetothermal heating, is the primary design objective. Their higher magnetic moment produces stronger magnetophoretic manipulation and mechanical actuation, making them attractive for magnetically actuated scaffolds, soft robotic systems, mechanically stimulated tissue constructs and guided implant positioning. This advantage, however limited by a remanent magnetization which promote aggregation, disrupt jet stability and fiber morphology [[98], [99], [100], [101], [102]]. Anisotropic particles such as nanocubes improve heating through shape-dependent anisotropy, although synthesis, alignment and batch reproducibility remain difficult [29,103]. Thus, increasing particle size or magnetic moments does not necessarily improve overall MREF performance.

Doped ferrites and metallic alloys offer stronger anisotropy, magnetostriction or heating, but these gains carry substantial safety costs. CoFe_2_O_4_ is valuable for magnetostrictive systems, whereas MnFe_2_O_4_ may support combined heating and imaging. However, cobalt-, nickel- and manganese-containing fillers require careful evaluation of ion release and cumulative exposure [[104], [105], [106], [107], [108], [109], [110]]. FeCo, FePt and Ni-based systems present additional concerns related to toxicity, oxidation, poor biodegradability and regulatory acceptability [104,[118], [119], [120], [121]].These materials are therefore difficult to justify for permanent implantation unless their function cannot be achieved with iron oxide.

Surface chemistry is equally important because poor particle dispersion can reduce the magnetic performance of even a well-selected filler. Oleic acid and organosilanes improve compatibility with hydrophobic polymers, whereas PEG, citrate and aminosilanes support dispersion or coupling in hydrophilic systems [100,122,123]. Amine functionalization can improve polymer coupling and field-driven transport [23], while polydopamine provides broad adhesion and secondary functionalization [122,124]. However, excessive coating, incomplete ligand exchange or aggregation can reduce effective magnetization and compromise fiber reproducibility [5,125,126]. Ligand density and particle size distribution should therefore be measured before electrospinning, and magnetic performance should be verified in the final fiber rather than inferred from the starting suspension.

Iron oxide has the strongest translational position, but its clinical precedent should not be interpreted as proof of MREF safety. Particle coating, loading, aggregation and release from the degrading polymer remain formulation specific [15,120]. Alternative ferrites and alloys face an even greater evidentiary burden because their degradation products may lack a benign metabolic pathway [[127], [128], [129]]. Our recommendation is unambiguous, iron oxide should remain the default filler for implantable MREFs, and any departure from iron oxide should be justified by a clear performance advantage and a corresponding biosafety and regulatory strategy.

Regulatory standing is strongly material dependent. Among the magnetic fillers considered for biomedical MREFs, iron oxides have by far the clearest clinical and regulatory precedent. Ferumoxytol is marketed in the United States as Feraheme® for iron-deficiency anaemia [130], while Ferabright®, based on the same iron oxide active ingredient, received FDA approval in 2025 for MRI of the brain in adults with known or suspected malignant neoplasms [131]. In Europe, the aminosilane-coated NanoTherm® system received CE marking in 2010 as a medical device for magnetic hyperthermia of brain tumors [18,132], establishing that an iron oxide-based therapeutic can be regulated through the device route; however, its previous CE marking should not be interpreted as a current authorization. Doped ferrites (CoFe_2_O_4_, MnFe_2_O_4_, NiFe_2_O_4_) and metallic fillers (FeCo, FePt, nickel-based materials) have no comparable clinical standing and would face a substantially greater evidentiary burden, because their degradation may release cobalt, nickel, manganese or other metal species whose long-term retention, systemic distribution and clearance remain poorly defined [104,106,[108], [109], [110],^118^,^133^].

Neither the FDA nor the European system provides a separate marketing-authorization pathway dedicated to nanomedicines; instead, nanomaterial-containing products are evaluated through existing drug, biologic, medical-device or combination-product frameworks. EMA nevertheless provides nanomedicine-specific scientific guidance within the medicinal-product framework [134,135], including a reflection paper requiring comparative quality, non-clinical biodistribution and human pharmacokinetic evidence for follow-on intravenous iron-based nanocolloids [136]. In the United States, a product combining drug and device functions is assigned to an FDA lead center by its primary mode of action [134]. European classification similarly depends on whether the principal intended action is pharmacological, immunological or metabolic or primarily achieved through a device-mediated, and a medicinal product incorporating an integral device may additionally require documentation under Article 117 of the Medical Device Regulation [137]. For MREFs, which integrate a degradable polymer matrix, a magnetic phase and frequently a therapeutic payload, uncertainty over drug, device or combination-product classification therefore represents an independent translational barrier.

A major weakness in the MREF literature is that magnetic filler is frequently unreported or reported ambiguously which prevents meaningful comparison. Loading should therefore be reported as wt.% of the total dry fiber, verified by thermogravimetric or elemental analysis, and accompanied by the saturation magnetization of the final composite fiber. Available data show that increasing filler content improves magnetic response only up to a formulation-dependent limit. Beyond this point, aggregation, jet instability, fiber defects, embrittlement, and uncontrolled release can outweigh the magnetic benefit [37,138]. Table 5 therefore highlights not only the loading used but also the point at which further loading became ineffective or detrimental.

**Table 5:** Magnetic filler loading in representative MREF studies.

Matrix	Magnetic filler	Reported or normalized loading	Main outcome	Interpretation	Reference
PCL	Iron oxide nanocubes	∼4.35 wt%*	Strong AMF heating with intrafiber magnetic chaining	Particle arrangement can be as important as total loading	29
PAN hybrid	Fe–Mn oxide nanoparticles	∼6–14 wt%*	5–7 emu g^−1^ magnetization with efficient AMF ΔT up to 8-12 °C	Efficient heating, but loading–heating proportionality was not established.	139
PCL–PNIPAAm	Fe_3_O_4_ nanoparticles	10 and 20 wt% ∼9 and 17 wt% by TGA	Temperature increase of approximately 5 and 8 °C	Doubling the magnetic loading increased heating sub-proportionally	140
CAP/PVA–PU	CoFe_2_O_4_	5 wt%	AMF triggered accelerated drug release	Moderate filler loading was sufficient for AMF-response release	43
PLLA	Fe_3_O_4_	2 and 5 wt%	Enhance bone regeneration at both formation	Biological benefit did not increase proportionally with loading	36
PLLA	Oleic-acid-coated iron oxide	0.5–8 wt% relative to polymer	6 wt% enabled rapid alignment; 8 wt% impaired fiber formation	Demonstrates a narrow functional processing window	13
PLLA	Fe_3_O_4_	4.0–11.1 wt%*	Magnetization increased from 1.88 to 4.42 emu g^−1^, while thermal stability decreased	Greater magnetic response was achieved at the expense of thermal stability	37
PBS	Fe_3_O_4_ nanopowder	∼5–10 wt%*; measured 2.4–3.8 wt%	Superparamagnetic behavior with NIR-triggered drug release	Final loading was substantially below the nominal value; sedimentation and incomplete incorporation during spinning limit magnetic loading.	41

Values marked with an asterisk were normalized from the reported dry formulation to facilitate comparison between studies.

These studies do not support a universal optimum such as 5–15 wt% [141]. The preferred loading is the lowest amount that produces the required heating, guidance, or mechanical response while preserving fiber morphology, mechanics, and release control. When this limit is reached, coaxial confinement or post-fabrication surface loading is preferable to further increase the blend concentration.

### 2.3 Functional components

MREFs increasingly incorporate functional components beyond the polymer-filler system. This section focuses on three functional additive classes including therapeutic payloads, electroactive components, and imaging probes. Each directly interacts with magnetic actuation by enabling triggered release, converting magnetostrictive deformation into electrical stimulation, or providing diagnostic contrast. Additives that only modify fiber processing or structure, such as plasticizers, porogens, and mechanical reinforcements, are treated as matrix modifiers in Section 2.1 rather than a functional component. Table 6 summarizes the functional components that directly support magnetically controlled release, electrical stimulation or imaging in MREFs.

**Table 6:** Functional components incorporated into MREFs.

Category	Representative components	Magnetic coupling	Main formulation issue	Main application	Refs
Small-molecule drugs	Doxorubicin, paclitaxel, curcumin, temozolomide, ketorolac	Heat-enhanced or thermally gated release	Burst release of hydrophilic drugs	Local cancer therapy; analgesia	[22,29,43,139]
Antimicrobials	Vancomycin, ciprofloxacin, rifampicin, Ag nanoparticles	Drug release combined with magnetothermal antibacterial action	Thermal safety; subtherapeutic dosing	Wounds; implant coatings	[30,67,142,143]
Proteins and growth factors	VEGF, BMP-2, NGF, TGF-β3, FGF-2	Magnetically enhanced release	Loss of bioactivity during processing	Bone, nerve and vascular repair	[57,144,145]
Nucleic acids	miRNA, siRNA, plasmid DNA	Triggered or staged release	Requires protection from solvents and degradation	Gene-activated scaffolds	[57,144]
Magnetoelectric components	CNTs, graphene oxide, PANI, PPy, PEDOT:PSS PVDF, P(VDF-TrFE), piezoelectric ceramics	Convert magnetostrictive strain into electrical cues	Requires effective mechanical coupling	Neural, cardiac and bone repair	[56,61,[146], [147], [148]]
Intrinsic MRI probes	Fe_3_O_4_, γ-Fe_2_O_3_	Magnetic filler provides T_2_/T_2_* contrast	Clustering and signal voids	Scaffold tracking	[25,31,149]
Secondary imaging probes	FITC, Cy5.5, gold nanorods,^89^Zr,^68^Ga	Adds optical, photoacoustic or nuclear imaging	Limited depth, stability or half-life	Multimodal theranostics	[25,31,150,151]

Therapeutic agents are among the most clinically significant functional components. Payloads include small-molecule chemotherapeutics (doxorubicin, paclitaxel, curcumin, cisplatin), antibiotics (vancomycin, ciprofloxacin, rifampicin), and anti-inflammatory drugs (ibuprofen, dexamethasone). Beyond pharmacological agents, bioactive proteins and growth factors (VEGF, BMP-2, NGF, TGF-β3, FGF-2) direct tissue specific regenerative signaling and nucleic acid payloads (miRNA, and plasmid DNA) extend MREF functionality towards gene-level control [57,144]. Chen et al. fabricated PCL nanofiber meshes incorporating MNPs, doxorubicin, and 17-AAG, a heat shock protein inhibitor. Under AMF exposure, these constructs reached hyperthermia temperatures while sustained release promoted synergistic apoptosis through combined hyperthermia, chemotherapy, and thermo-molecularly targeting [152]. This design is instructive because it addresses a failure mode intrinsic to magnetothermal therapy in which heat shock protein mediated thermotolerance reduces the efficacy of repeated hyperthermia. By incorporating a payload that counteracts this response, the design illustrates a key MREF principle in which the payload complements the actuation mechanism rather than only serving as cargo.

Electroactive additives, including carbon nanotubes (CNTs), graphene oxide (GO), gold nanowires, and conductive polymers such as polyaniline (PANI), poly(3,4-ethylenedioxythiophene) (PEDOT) and polypyrrole (PPy), are incorporated with magnetic fillers can improve the electrical conductivity of cardiac and neural scaffolds, where passive polymers alone may not support effective electrophysiological coupling [[146], [147], [148]]. In support of this rationale, Zhou et al. demonstrated that CNT incorporation improves cardiac-cell adhesion, proliferation, electrophysiological maturation, and excitation–contraction coupling [153]. When combined with Fe_3_O_4_ or γ-Fe_2_O_3_ in PU or PGS fiber matrices, the conductive phase (CNTs, GO, PANI, or PPy) supports electrical conductivity and mechanical reinforcement, whereas the magnetic phase provides heating, magnetic guidance or mechanical actuation.

Imaging probes further evaluate MREFs into multimodal theranostic platforms. Iron oxide can provide both magnetic actuation and T_2_/T_2_*-weighted MRI contrast, while fluorescent labels (FITC-labeled polymers, Cy5.5-conjugated**)**, gold nanorods and radiolabels such as ^89^Zr and ^68^Ga add optical, NIR or nuclear imaging [150,151]. Their value lies in confirming implant placement and monitoring persistence, although additional labels increase formulation and regulatory complexity. Tissue-instructive components such as hydroxyapatite, collagen, gelatin, RGD peptides and laminin improve adhesion, mineralization, lineage-specific differentiation and integration [26,154]. Qian et al. fabricated core-sheath PCL nanofibers with simvastatin in the core and nanohydroxyapatite on the surface, achieving prolonged release and improved proliferation in bone-regenerative scaffolds [155]. Together, these additives support the development of MREFs as theranostic platforms rather than passive scaffolds. The same integration, however, makes performance difficult to attribute and concentrates safety and reproducibility concerns in a more complex composite. Each added component should therefore provide a distinct function that cannot be achieved by the magnetic fiber alone.

## 3 Fabrication strategies

**Fig. 1:**
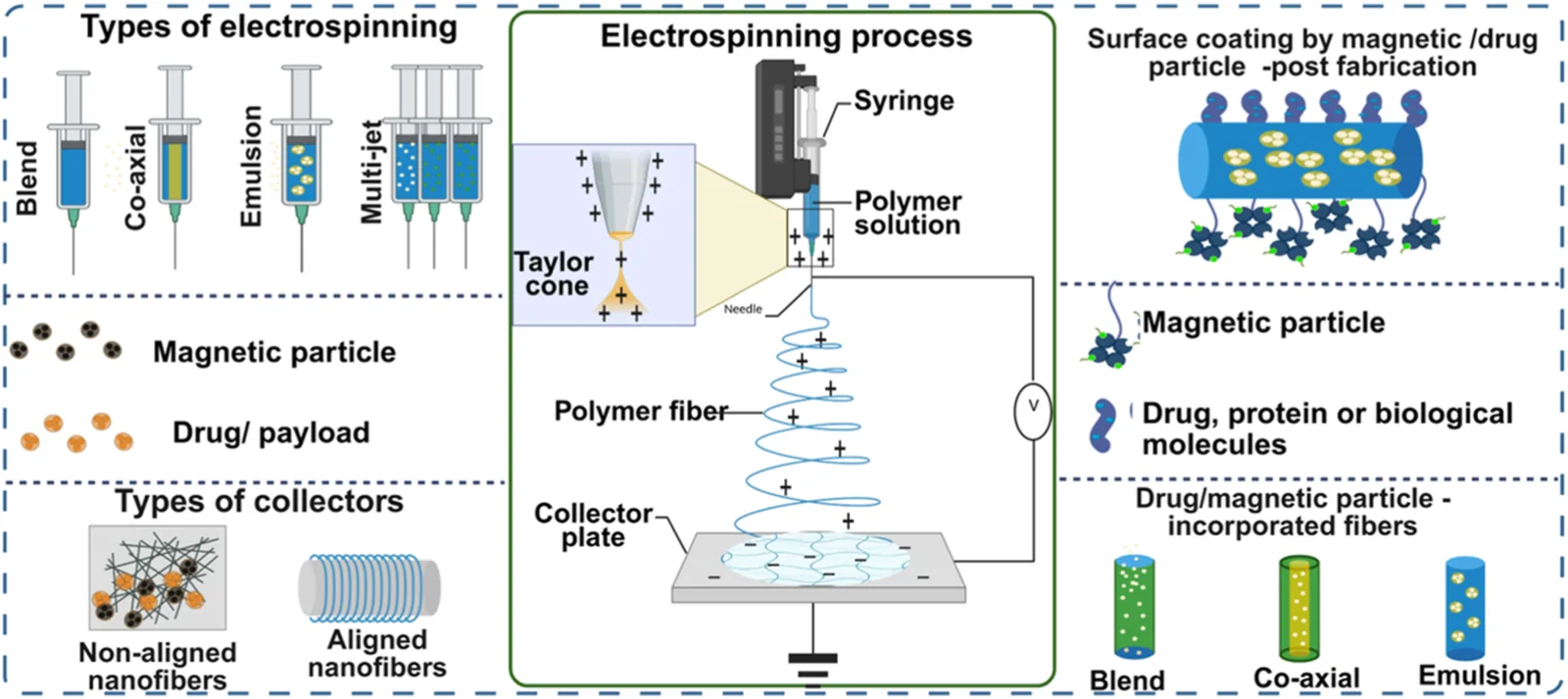
The schematic illustrates major fabrication approaches used to incorporate magnetic particles, drugs, proteins, or biological molecules into electrospun fibrous systems.

Electrospun fibers are produced through jet elongation and solvent evaporation [156]. As illustrated in Fig. 1, MREF fabrication can be achieved using different electrospinning configurations, followed by either direct incorporation or post-fabrication surface coating with magnetic particles, drugs, proteins, or biological molecules. Electrospinning provides control over fiber diameter, porosity, surface-to-volume ratio and fiber architecture. These features are highly process-dependent and are not independent design variables. Reported fibers mats commonly exhibit porosities of approximately 60-90%, and fiber diameters ranging from tens of nanometers to several micrometers [[157], [158], [159]]. The critical challenge is therefore not simply achieving a desired morphology, but maintaining it after magnetic filler addition, which can alter solution viscosity, conductivity, jet stability and particle distribution. Process optimization should consequently be evaluated because they control burst release, diffusion rate, matrix wettability, mechanical compliance and cell–material interactions, rather than morphology alone.

### 3.1 Blend electrospinning

Blend electrospinning is the simplest and most common approach for MREF fabrication where magnetic fillers are dispersed directly into the polymer solution before spinning, either alone or with therapeutic agents, conductive fillers or secondary nanocarriers. Its advantages are operational simplicity, compatibility with existing single-needle setups, and the ability to co-load therapeutic agents with fillers without additional synthetic steps. However, drug encapsulation efficiency is formulation-dependent and is governed by drug–polymer compatibility, solvent selection and nanoparticle dispersion rather than by a universal magnetic-loading threshold (approximately 10–15 wt% of total dry fiber mass) [141,160].

Blend electrospinning also has important limitations. All components must remain compatible with a common solvent system, limiting the incorporation of hydrophilic payloads and solvent-sensitive biologics [161,162]. Hydrophilic drugs migrate toward the fiber surface during jet stretching and solvent evaporation, producing burst release that often exceeds 40% within the first 24 h^163^. Increasing magnetic filler content beyond approximately 10–15 wt% further destabilizes the jet through increased viscosity and particle aggregation, resulting in broader fiber-diameter distributions, bead formation and reduced reproducibility [5,163,164]. Labile biologics such as proteins, growth factors and nucleic acids are also susceptible to denaturation or degradation during exposure to organic solvents and high electric fields [165].

Despite these limitations, blend electrospinning is therefore most appropriate for hydrophobic small-molecule formulations where moderate magnetic loading is sufficient and precise spatial compartmentalization is unnecessary. More complex payloads requiring protection or staged release are generally better served by compartmentalized architectures such as coaxial or emulsion electrospinning. Studies by Ziegler et al. [139]., and Radoń et al. [166]. demonstrate that careful control of particle dispersion, surface chemistry, solvent composition and filler loading can produce reproducible MREFs with controlled morphology, efficient magnetic heating and field-responsive drug release, but these outcomes depend on formulation optimization rather than the blend-spinning process itself.

### 3.2 Core–shell and hierarchical architectures

Coaxial electrospinning overcomes the shared-solvent limitation by delivering two solutions through concentric needles, forming a compound fiber in which the inner phase is enclosed by an outer sheath. Its main advantage is protection of hydrophilic drugs, proteins, growth factors from organic solvents, while the shell limits premature diffusion [167,168]. This control however depends on narrow operating window governed by solution and process parameters, including core/sheath viscosity ratio, miscibility, conductivity, interfacial tension, and flow-rate ratio. Although the viscosity ratio of approximately 1.22–2.82^170^ and core-to-shell flow-rate ratio, near 1:4–1:6 have produced stable fiber in selected systems [169,170]. In optimized coaxial systems, small molecule drugs can reach encapsulation efficiencies of 94-99%, while proteins are encapsulated with high loading efficiency and preserved bioactivity [171]. The shell reduces burst release to about 10–15% or lower and increases loading capacity compared to blend electrospinning at equivalent polymer concentrations [[171], [172], [173]]. However, these values are formulation-specific and should not be treated as universal performance benchmarks.

The positioning of magnetic filler within coaxial fibers enables distinct functional configurations. A filler-containing shell can generate heat close to the diffusion barrier, whereas placing the core-filler adjacent to a thermoresponsive shell compartment improves thermal gating. Thermoresponsive core–shell fibers, such as PCL/PEG–PNIPAAm or PCL/PNIPAAm systems, demonstrate that a responsive shell can regulate drug diffusion through temperature-dependent swelling, providing the design rationale for incorporating magnetic fillers into future coaxial MREFs [174,175]. Triaxial electrospinning further extends this concept to radially graded fibers with three concentric compartments enabling sequential release of multiple agents form spatially separated regions [176]. Its value is greatest where sequential biological cues are required, such as inflammation control followed by angiogenesis or regeneration. Nevertheless, the added architectural freedom comes with lower throughput, narrower processing window and greater quality-control requirements, which remain major barriers to clinical scale-up. Triaxial designs should therefore be reserved for applications in which multistage release provides a clear advantage over simpler coaxial or blend fibers.

### 3.3 Emulsion and other electrospinning approaches

Emulsion electrospinning encapsulates hydrophilic biologics within hydrophobic matrices by dispersing an aqueous phase as droplets within a continuous organic polymer/filler phase, limiting exposure of sensitive payloads to organic solvents [177]. Its performance depends strongly on emulsion stability because droplet size, controlled by surfactant chemistry, concentration, and homogenization determines internal compartment formation, fiber morphology and release kinetics [178]. The method therefore offers payload protection with a single nozzle, but less precise compartmentalization and greater batch sensitivity than coaxial electrospinning. Janus electrospinning creates two chemically distinct side-by-side domains within a single fiber, enabling asymmetric payload placement and bidirectional interfacial properties [[179], [180], [181]]. This architecture is potentially useful at tissue boundaries such as periosteum–bone or dura–neural interfaces, although maintaining a stable interface and reproducible domain geometry remains a significant manufacturing challenge.

### 3.4 Post-fabrication functionalization

Post-fabrication functionalization modifies the surface of electrospun fibers without altering the bulk fiber matrix. Common approaches include plasma treatment, wet-chemical modification, covalent grafting and layer-by-layer deposition which can increase surface reactivity, improve wettability and introduce binding sites for drugs, proteins or recognition ligands. Oxygen, nitrogen, or argon plasma treatment can reduce the water contact angle of PCL fibers from approximately 120–135° to below 50°, and in some optimized systems to near 0° [[182], [183], [184], [185]]. Coating based in APTES, dopamine, or poly-L-lysine provide secondary reaction sites for biomolecule immobilization and improve cell-material recognition [[186], [187], [188], [189]] whereas, layer-by-layer assembly enables nanoscale control over surface charge, payload deposition and degradation [190]. However, these improvements are highly process-dependent and may be transient if surface rearrangement, coating delamination or ligand leaching occurs.

For MREF, post-fabrication surface decoration provides an alternative to blend incorporation, because magnetic particles can be concentrated at the fiber surface without destabilizing the electrospinning jet or exposing internal payloads to the particles [22,189]. This configuration may improve surface-localized heating and protect thermolabile cargo, but it sacrifices particle retention. Surface-bound fillers are more susceptible to detachment under physiological flow, deformation and degradation than embedded particles [191,192]. Post-fabrication decoration is therefore most appropriate when surface accessibility is functionally necessary, and its translational use should require direct testing of coating uniformity, magnetic performance and particle retention under application-relevant conditions.

### 3.5 Comparative evaluation of fabrication routes

No single fabrication route is optimal for all MREF applications. Blend electrospinning offers the simplest and most scalable process but provides limited control over filler distribution and payload release [156,166]. Coaxial and triaxial architecture provide better compartmentalization, payload protection and burst release control, although their coupled flow conditions, narrower operating windows and lower throughput complicate reproducible scale-up [167,176,193]. Emulsion electrospinning provides partial compartmentalization using a single nozzle, but its performance depends strongly on emulsion stability during processing [177,178]. Post-fabrication functionalization avoids exposing labile agents to organic solvents and high voltage, but surface-bound particles or payloads may detach during washing, implantation or matrix degradation [186,189,194].

Table 7 highlights a central design conflict between functional control and translational feasibility. Route selection should be determined by the actuation mechanism, payload stability, filler retention, and manufacturing pathway rather than by release performance alone. Blend electrospinning is most appropriate when scalability, embedded-particle retention and moderate magnetic loading are priorities. Coaxial and triaxial systems are justified when proteins or thermoresponsive components require spatial protection or staged release. Post-fabrication modification is preferable for surface-localized functions or processing-sensitive payloads, provided coating uniformity and retention are demonstrated. A hybrid strategy that embeds the magnetic filler during electrospinning and adds the labile payload afterward may offer the most practical balance between magnetic-particle retention, payload bioactivity and manufacturing scalability.

**Table 7:** Comparative evaluation of MREF fabrication routes.

Criterion	Blend electrospinning	Coaxial electrospinning	Triaxial electrospinning	Emulsion electrospinning	Post-fabrication decoration
Operational complexity	Low; single feed	Moderate; concentric nozzle and two feeds	High; three feeds and narrow operating window	Moderate; requires emulsion stability	Low–moderate; adds a modification step
Hydrophobic small molecules	High compatibility	High compatibility and release control	High; supports staged release	Limited advantage over blend spinning	Limited to surface loading
Hydrophilic small molecules	Low; surface migration and burst release	High; protected core	High; strong compartmental control	High; aqueous-phase encapsulation	Low; rapid surface release
Proteins and growth factors	Low; direct solvent exposure	High; core protects bioactivity	High; suitable for staged release	Moderate–high; dependent on emulsion stability	High; mild aqueous immobilization
Nucleic acids	Low; limited protection	High; protected core	High; staged delivery possible	Moderate; emulsification may damage cargo	Moderate; requires stable conjugation
Magnetic loading	Moderate; limited by dispersion and jet stability	High effective loading through compartmentalization	High architectural flexibility	Moderate; similar constraints to blend spinning	High surface loading without jet disruption
Filler localization	Low	High; core or shell	Very high; multiple compartments	Low–moderate	Very high; surface only
Burst-release control	Low	High	Very high	Moderate	Low for surface-loaded payloads
Filler retention	High when embedded	High	High	High	Lower; surface loss requires testing
Scalability	High; compatible with multi-needle production	Low–moderate	Low	Moderate; long-run emulsion stability is limiting	Moderate–high; adds washing and quality control
Main translational risk	Batch-dependent dispersion	Manufacturing complexity	Poor manufacturability	Emulsion drift	Non-uniform coverage and filler shedding
References	[37,141,156,160,166]	[167,168,173,193,[195], [196], [197]]	[171,176]	[177,178]	[22,[182], [183], [184],[186], [187], [188], [189], [190],^194^,^198^]

Note: Comparative ratings represent a qualitative synthesis of the cited literature. Scalability reflects a critical synthesis of process complexity and demonstrated production routes rather than direct head-to-head manufacturing studies.

## 4 Mechanism of magnetic action in biomedical settings

**Fig. 2:**
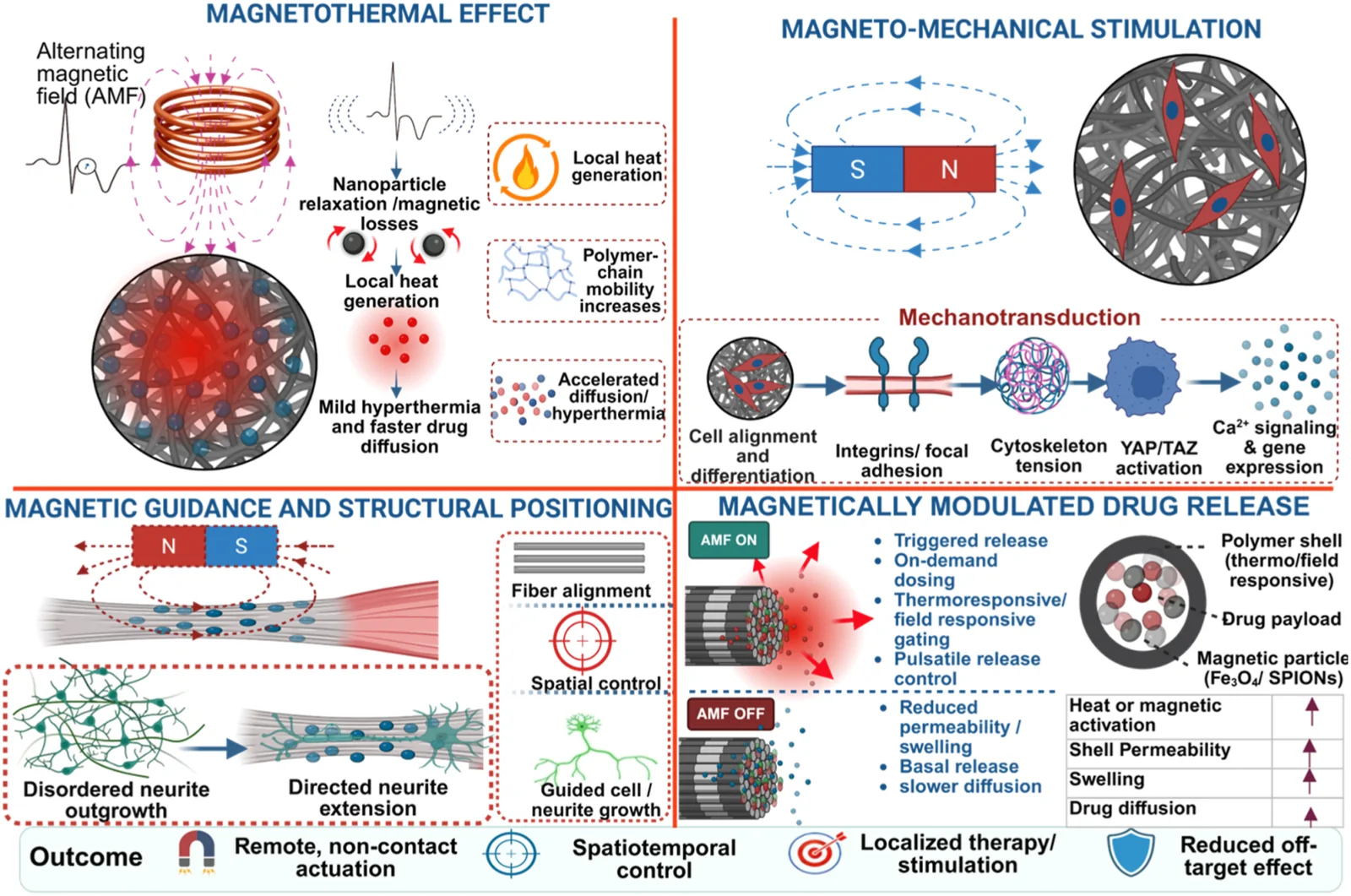
Mechanisms of magnetic action in magnetic-responsive electrospun fibers (MREFs). MREFs can respond to external magnetic fields through magnetothermal heating, magneto-mechanical stimulation, magnetic guidance, and AMF-controlled drug release. These mechanisms enable remote non-contact actuation, spatiotemporal control, localized therapy or cell stimulation, and reduced off-target effects in biomedical applications.

Magnetically responsive electrospun fibers operate through three primary physical pathways including magnetothermal heating, magneto-mechanical force or torque, and magnetic guidance. Magnetically modulated drug release is not a separate mechanism but a functional outcome of these three pathways. Release is most driven by heating, although low-frequency mechanical actuation can also trigger release without a measurable temperature increase, as demonstrated by Funnell et al. [145]. Fig. 2 summarizes these mechanisms and their associated release responses.

### 4.1 Magnetothermal effect

The magnetothermal effect is conversion of AMF energy into localized heat by magnetic fillers embedded in the fiber matrix. Under AMF, the particles continuously attempt to realign with the oscillating field and dissipate energy into heat that can be used for hyperthermia, thermally accelerated diffusion, polymer swelling or thermoresponsive gating [17,99,199,200].

Superparamagnetic particles dissipate energy mainly through Néel relaxation after fiber immobilization because Brownian rotation is strongly restricted [99,199,200]. Consequently, SAR measured in ferrofluids can substantially overestimate heating in the final scaffold. Blocked single-domain particles instead generate hysteretic losses, but their remanence promotes aggregation during spinning and can compromise fiber uniformity. Larger multidomain particles offer higher magnetic moments but are not necessarily better heaters, because domain-wall losses may remain modest at clinically acceptable field amplitudes. Their main value is therefore magnetic force and mechanical actuation rather than hyperthermia. Table 7 is therefore functionally important because particles of the same composition can require different heating models and present different processing risks.

This mechanistic dependence makes cross-study comparison difficult. SAR and SLP values are meaningful only when reported with field amplitude, frequency, filler mass, scaffold mass and measurement medium. The condition $2\pi f\tau_{\text{eff}}\approx 1$ provides a useful design principle, but electrospinning changes $\tau_{\text{eff}}$ (effective relaxation time) through immobilization and particle interactions. Heating should therefore be measured directly in the final fibrous construct rather than inferred from nanoparticle suspensions [199,201].

AMF exposure is also constrained by non-specific tissue heating. The Atkinson–Brezovich product, $Hf\leq 4.85\times 10^{8}\;A\;m^{-1}\;s^{-1}$, is widely cited but should not be treated as a universal limit because tolerability depends on coil geometry, exposed volume, perfusion and treatment duration [19,[201], [202], [203]]. Studies should report H, f, H·f, exposure time and treatment geometry explicitly. A poorly heating scaffold cannot be made clinically relevant simply by increasing the field.

Thermally driven release depends on whether local heating is sufficient to alter polymer mobility, water uptake, diffusion or a thermoresponsive transition. This response is therefore controlled by filler distribution, payload location and matrix architecture, not by temperature alone. Mild hyperthermia between approximately 39 and 45 °C may also sensitize tumor cells to chemotherapy and radiotherapy [18,204]. However, many studies report only bulk temperature rise and cumulative release, without resolving local fiber temperature, reversibility or repeated-cycle performance.

Representative studies support the feasibility of magnetothermal release but also expose the lack of standardization. Ziegler et al. reported temperature increases of approximately 8 °C during a 5 min AMF pulse, with AMF-triggered of hydrophilic ketorolac and hydrophobic curcumin release where the strong response occurs when the drugs were loaded in mesoporous silica nanoparticles embedded within the fibers [139]. Other systems reached hyperthermic temperatures or enabled thermoresponsive ON–OFF release [[205], [206], [207]]. These results cannot be ranked directly because field conditions, filler loading, sample geometry and thermal environment differ. The critical evidence gap is therefore not whether MREFs can heat, but whether they can deliver reproducible, spatially controlled and tissue-safe heating under clinically realistic conditions. Fernández et al. reported enhanced acetaminophen diffusion from PVA/Fe_3_O_4_ fibers under a 0.5 T static magnetic field [46]. Because static fields do not produce conventional magnetothermal heating, the response was likely associated with field-induced particle–polymer interactions or changes in diffusion pathways. Nevertheless, without direct measurements of fiber deformation, force or local temperature, this result should be interpreted as non-thermal magnetic modulation.

### 4.2 Magneto-mechanical stimulation

Magneto-mechanical stimulation occurs when magnetic fillers convert magnetic energy into localized mechanical outputs such as forces, torque, bending, vibration, or scaffold deformation. These cues influence cell behavior by regulating cell spreading, cytoskeletal organization, adhesion maturation, and lineage-specific differentiation. The magnitude of the responses is governed not only by the filler but by the loading, dispersion, fiber diameter, polymer stiffness, fiber alignment, and the strength, direction and geometry of the field. Magneto-mechanical stimulation is therefore not an intrinsic property of the nanoparticle but an integrated response of the filler–matrix–cell interface [208,209].

At the cellular level, deformation of cell-scaffold interface activates integrins and focal adhesion complexes, increases cytoskeletal tension and initiates downstream effectors (FAK, RhoA/ROCK, YAP/TAZ, MAPK/ERK, Wnt/β-catenin, BMP/Smad, and Ca^2+^-dependent signaling). Mechanosensitive ion channels e.g. Piezo1, translate membrane tension into calcium influx and transcriptional responses. These mechanisms are most relevant in load bearing or mechanically dynamic tissues such as bone, tendon, skeletal muscle, vasculature, and peripheral nerve [208,210,211].

Magneto-mechanical stimulation offers a non-invasive route to delivering mechanical signals without wired devices, or external loading systems. Magnetic scaffolds can enhance osteogenesis and promote tenogenic mechanotransduction, reproduce contractile-like stimulation in muscle models and guide cell alignment and maturation in neural or vascular tissues [212,213]. Tomás et al. incorporated magnetic nanoparticles into tendon-mimetic fibrous scaffolds and showed that remote magnetic actuation promoted anisotropic cytoskeletal organization in human adipose-derived stem cells, modulated the mechanosensitive YAP/TAZ pathway and increased tendon-related markers expression [214].

The central limitation of this field is therefore stimulus quantification. Local force and strain vary across the scaffold with particle distribution, fiber diameter, matrix stiffness and cell position, yet most studies report only downstream biological responses such as YAP/TAZ activation or marker expression. This prevents meaningful comparison between studies and limits translation to larger constructs. Mechanistic attribution is further weakened by inadequate controls. Studies should therefore report estimated force or strain and compare magnetic fibers with non-magnetic fibers under the same field and with magnetic fibers exposed to an equivalent thermal profile. Until these controls are standard, claims of magneto-mechanical action should remain provisional [215,216]. Increasing filler loading does not necessarily solve this problem because higher magnetic forces may be offset by reduced porosity and scaffold deformability.

### 4.3 Magnetic guidance and structural positioning

Magnetic guidance describes the ability of MREFs to be spatially directed, aligned, retained, or reorganized by an external magnetic field using field gradients and magnetic torque to control the location and orientation. The fibers provide a topographical cue while fillers allow those cues to be positioned or oriented remotely after fabrication [200,215].

Magnetic guidance can function through two primary mechanisms. First, field gradients direct magnetic particles or fiber fragments toward a target site, increasing local retention. Second, fields organize the internal architecture of the scaffold by aligning fibers, orienting magnetic domains or positioning injectable fiber segments within a hydrogel matrix [217]. Liu et al. demonstrated that an external magnetic field at the collector generates aligned and wavy biodegradable fibers and that these aligned matrices promote elongated stem-cell morphology along the fiber axis [218]. Johnson et al. developed injectable, magnetically oriented PLLA fiber conduits containing SPIONs that could be injected into a hydrogel and then manipulated *in situ,* with aligned fibers providing directional guidance to neurons. This study shows that MREFs can function as minimally invasive, magnetically positional guidance structures rather than pre-formed implanted mats [13]. Hiraki et al. embedded SPIONs in fiber segments and aligned them within a three-dimensional hydrogel, tuning the degree of alignment by adjusting SPION density and field strength [219].

The principal limitation of magnetic guidance is the rapid decay of field gradients with distance. Magnetophoretic force depends on magnetic volume, particle magnetization and the field gradient rather than field strength alone. At depths greater than a few centimeters, clinically practical external magnets generally provide insufficient gradients for reliable targeting. Magnetic guidance of MREFs is therefore most credible for superficial, surgically exposed or endoluminal sites, where the magnetic source can be positioned close to the construct, as in the stent system reported by Aguilar et al. [207]. This constraint is imposed mainly by the field source and cannot be fully overcome by increasing particle performance. Claims of deep tissue magnetic targeting should therefore be interpreted cautiously.

## 5 Biomedical applications

The biomedical applications of MREFs are summarized in Table 8 which compares polymer selection, magnetic filler type, therapeutic payload, and magnetic trigger method to show how MREFs designs are tailored to each application. These examples highlight the versatility of MREFs as locally retained, magnetically activatable platforms for controlled release, hyperthermia, tissue guidance, antimicrobial activity, imaging, and sensing.

**Table 8:** Representative MREFs systems for biomedical applications.

Application	System	Polymer selection	Magnetic filler	Payload	Trigger method	References
Cancer therapy	Implantable smart magnetic nanofiber device	PLGA-based nanofibrous implant	Magnetic nanoparticles	Bortezomib	AMF hyperthermia + pH-sensitive release	220
	Injectable hyperthermic nanofiber mesh	Thermoresponsive nanofiber mesh	Magnetic nanoparticles	Paclitaxel	AMF-triggered heating and release	54
	Magnetic nanofiber bandage for skin cancer	PCL nanofibrous bandage	Fe_3_O_4_ nanoparticles	No drug; hyperthermia-focused	AMF-induced magnetic hyperthermia	221
	Smart hyperthermia nanofiber platform	PCL nanofiber mesh	Magnetic nanoparticles	Doxorubicin + 17AAG	AMF hyperthermia + sustained release	152
	DOX/iron oxide nanocube PCL fibers	PCL	Iron oxide nanocubes	Doxorubicin	Magnetic hyperthermia	29
	Core–shell lung-cancer nanofibers	CAP/PVA core and polyurethane shell	CoFe_2_O_4_ nanoparticles	Doxorubicin	AMF hyperthermia + pH-sensitive release	43
	pH-sensitive postsurgical breast-cancer nanofibers	PCL	Iron oxide nanoparticles	Doxorubicin + ammonium bicarbonate pH-responsive component	Acidic pH + AMF hyperthermia	74
Tissue engineering and regenerative medicine	Silk fibroin magnetic electrospun fibers	Silk fibroin	Fe_3_O_4_ or CoFe_2_O_4_ nanoparticles	No drug; scaffold platform	Magnetic responsiveness	69
	SPION-grafted aligned PLLA fibers	PLLA	Oleic-acid-coated iron oxide nanoparticles	No drug; neurite guidance scaffold	Static magnetic-field orientation	222
	Magnetic silk-fibroin cardiac nanofibers	Silk fibroin	SPION–casein core–shell nanoparticles	Embryonic cardiac cells	Magnetic scaffold functionality	223
	Aligned electrospun for peripheral nerve regeneration	PCL/Fe_3_O_4_ nanofibers followed by polypyrrole (Ppy) coating	Fe_3_O_4_ magnetic nanoparticles	No drug; conductive/magnetic nerve-regeneration scaffold	Static magnetic-field stimulation	61
	PCL/collagen/Fe_3_O_4_ bone scaffold	PCL/collagen type I	Fe_3_O_4_ nanoparticles	ADSCs/no drug	Magnetic scaffold cue	65
	SPION-based magnetic PLGA nanofibers	PLGA	SPIONs	MSCs/no drug	Magnetic scaffold cue	224
	CoFe_2_O_4_–PVDF magnetoelectric nanofiber patch	PVDF	CoFe_2_O_4_ nanoparticles	No drug; cardiac stimulation scaffold	Magnetic/mechanical/electrical activation	56
	Magnetic scaffold for osteochondral repair	Electrospun gelatin nanofiber-based 3D scaffold	Magnetic nanoparticles	MSCs	Rotating magnetic field	225
Wound healing and antimicrobial dressing	Chitosan/gelatin/Fe_3_O_4_ antibacterial membrane	Chitosan/gelatin	Fe_3_O_4_ nanoparticles	No drug; antimicrobial scaffold	Passive antibacterial/magnetic composite effect	67
	Ag-doped magnetite nanofibrous	PCL	Ag-doped magnetite nanoparticles	antibacterial Ag-doped MNPs	Passive antibacterial/magnetic composite effect	30
	CQDs–Fe_3_O_4_–rosemary electrospun dressing	PVA/cellulose nanofibrils	CQDs–Fe_3_O_4_ hybrid	Rosemary extract	Passive release	48
	CoFe_2_O_4_@CTAB/PVDF magnetoelectric dressing	PVDF	CoFe_2_O_4_@CTAB	No conventional drug; CTAB provides antibacterial function	Wearable dynamic magnetic field	143
	Pectin–PVA/Fe_3_O_4_/*Ficus carica* nanofiber dressing	Pectin/PVA	Fe_3_O_4_ nanoparticles	*Ficus carica* extract	Passive antioxidant/antibacterial release	73
	ZnO_2_/Fe_3_O_4_ double-layer catalytic dressing	Double-layer electrospun nanofibers	ZnO_2_ and Fe_3_O_4_ nanoparticles	Catalytic antibacterial components	H_2_O_2_/acid self-supplying catalytic therapy	226
	Fe_3_O_4_-loaded shape-memory polyurethane electrospun scaffold	Shape-memory polyurethane	Fe_3_O_4_ nanoparticles	No conventional drug	Magnetic/thermal shape-memory activation	44
	Alined PLLA/CoFe_2_O_4_ magneto-mechano-electric nanofibrous membrane	Aligned PLLA	CoFe_2_O_4_ nanoparticles	No drug; biophysical stimulation scaffold	External magnetic field	227
Theranostics and biosensing	Theranostic 3D nanofiber brain implant	PLGA/PLA/PCL nanofiber blend	Fe^2+^-doped calcium phosphate nanoparticles	Temozolomide	Sustained local release + MRI monitoring	149
	Implantable magneto-responsive poly(aspartamide) scaffold	Poly(aspartamide)-based electrospun mesh	Magnetite/MIONPs	No conventional drug; hyperthermia scaffold	AMF hyperthermia + MRI contrast	228
	PDA-coated nanocomposite theranostic implant	PCL/PLGA coaxial electrospun fibers	SPIONs	Methotrexate-loaded LDH	Sustained chemotherapy + MRI contrast	31
	Electrospun magnetic PU/Fe_3_O_4_ nanomesh with functionalized PEDOT	Magnetic polyurethane nanomesh with conductive PEDOT-based sensing layer	Fe_3_O_4_ nanoparticles	Biosensing elements for electrochemical detection	External magnetic-field deformation + electrochemical sensing	229
	Magnetic-particle-induced carbon nanofiber sponge sensor	Electrospun carbon nanofiber sponge/graphene composite	Magnetic particles	No drug; pressure-sensing platform	Mechanical pressure sensing	230
	Fe_3_O_4_/carbon nanofiber pressure sensor	3D electrospun carbon nanofibers	Fe_3_O_4_ nanoparticles	No drug; sensing platform	Piezoresistive pressure sensing	231
	Magnetic nanofiber for sensors	PAN-derived nanofibers	Fe_3_O_4_ nanoparticles	No drug; sensor material	Sensor platform development	232

### 5.1 Cancer therapy and magnetic hyperthermia

The most plausible clinical use of MREFs in oncology is locoregional treatment of a surgical resection cavity or an accessible unresectable tumor. Fibrous implants could be placed during surgery or delivered endoscopically or percutaneously. Carmustine wafers (Gliadel®), provide a relevant clinical benchmark, demonstrating biodegradable intracavitary implants can be safely positioned along a glioma resection cavity during surgery and achieve localized chemotherapy, although with only modest survival benefit [233,234]. However, magnetic fibers are justified only when they provide a clear advantage over passive implants. Their potential value lies in enabling post-implantation control of drug release, delivering hyperthermia as an independent cytotoxic and treatment-sensitizing modality, and permitting MRI-based localization or monitoring [31,220,235,236]. When these functions are not required or are not experimentally demonstrated, conventional non-magnetic drug-loaded fibers are the more rational and less complex design.

**Fig. 3:**
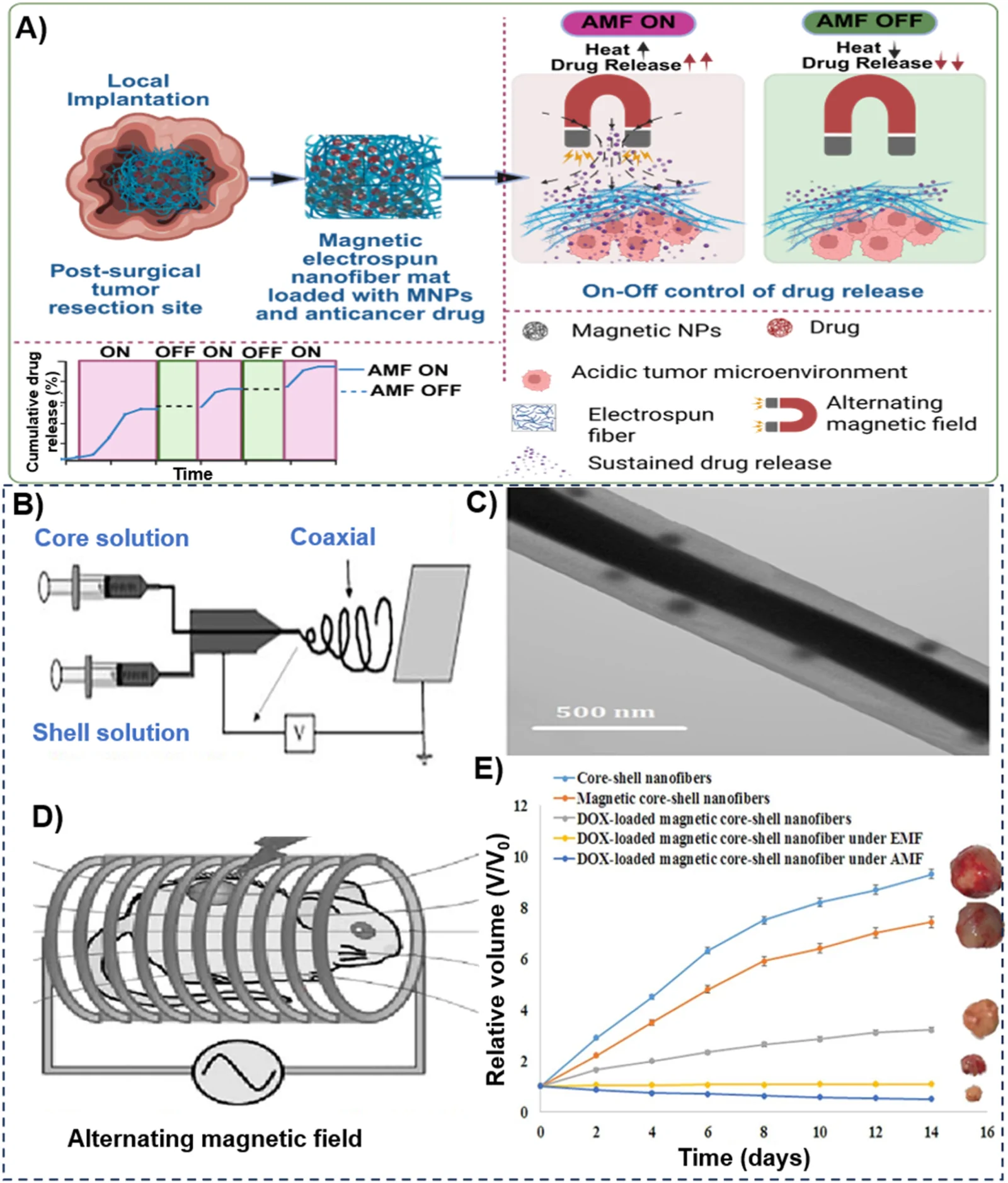
AMF-responsive magnetic electrospun nanofibers for localized post-surgical tumor therapy. (A) Schematic illustration showing local implantation of a magnetic electrospun nanofiber mat loaded with magnetic nanoparticles and anticancer drugs at the post-surgical tumor resection site, enabling heat-assisted, on–off controlled drug release under alternating magnetic field (AMF) stimulation. (B–E) Coaxial fabrication and evaluation of CAP/PVA-core–PU-shell fibers containing doxorubicin and CoFe_2_O_4_ nanoparticles. TEM image confirm fabrication of magnetic core–shell nanofibers, which demonstrate DOX-loaded magnetic core–shell nanofibers under AMF stimulation, achieve the strongest tumor-growth inhibition compared with the control formulations [43].

Locoregional therapy concentrates cytotoxic agents at the disease site while reducing dose-limiting systemic toxicity. In MREF-based oncology, the principal translational advantage is therefore not magnetic targeting after systemic administration, but local retention of a fibrous depot that can be reactivated after implantation. The rationale is important because passive nanoparticle accumulation through the EPR-effect remains inefficient, for which a median of 0.7% of the injected dose reach solid tumors [22,25,237]. As illustrated in Fig. 3A, magnetic nanofiber mats could be positioned within or adjacent to a resection cavity, where magnetic particles provide AMF-induced heating and on/off control of drug release. The additional complexity of the magnetic phase is justified only when these functions improve treatment beyond a passive local implant.

Serio et al. co-loaded doxorubicin and 23 nm iron oxide nanocubes into PCL fibers [29]. The intrafiber particle chaining enhanced magnetic heating, and AMF exposure increased cytotoxicity against cervical cancer cells through the combined effect of hyperthermia and drug release. This study demonstrates that particle organization within the fiber can be as important as nominal loading. However, its translational relevance remains dependent on whether comparable heating can be achieved in perfused tissue at clinically realistic implant masses and field conditions.

Bahmani et al., fabricated cellulose acetate phthalate CAP/PVA core and PU shell containing doxorubicin and CoFe_2_O_4_ nanoparticles (Fig. 3B–E) [43]. AMF exposure shortened the release period from approximately 300–600 h to 168–360 h, depending on pH, and the optimized construct killed more than 98.3 ± 0.2% of A549 cells *in vitro*, and with *in vivo* treatment. The core–shell architecture provides a convincing route for separating drug and magnetic functions, but the use of CoFe_2_O_4_ introduces cobalt-release and long-term safety concerns. This design is therefore more compelling as proof of magnetically controlled release.

**Fig. 4:**
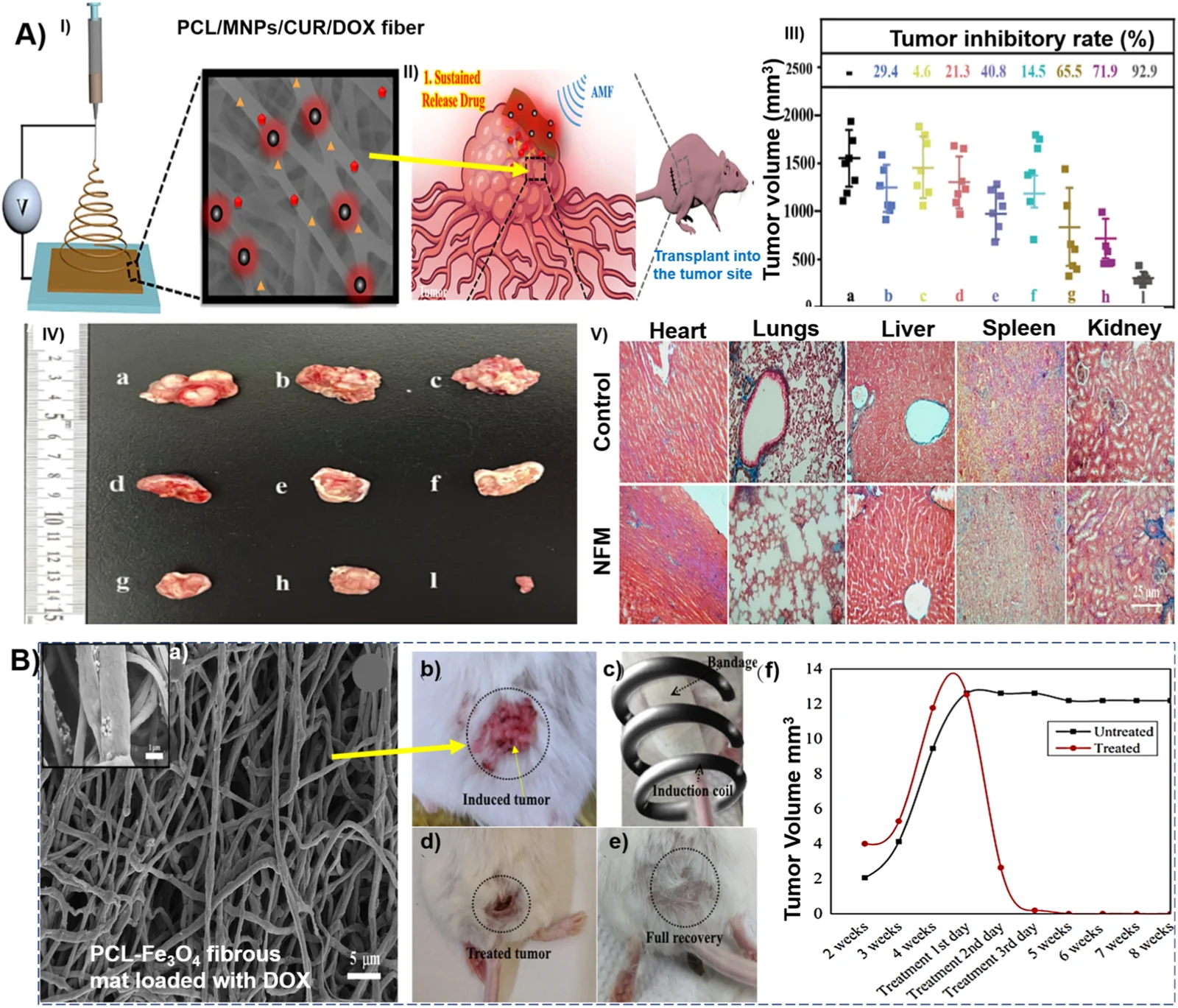
Magnetic electrospun fibers for localized tumor therapy and AMF-triggered drug delivery. (A) Schematic illustration of PCL nanofiber loaded with magnetic nanoparticle (MNPs) for delivering CUR and DOX. I) Schematic of the electrospinning process used to fabricate the PCL/MNPs/CUR/DOX nanofibrous mat, II) PCL/MNPs/CUR/DOX electrospun fibers were locally transplanted into the tumor site for sustained drug release and AMF-triggered magnetic activation demonstrating III) strong tumor inhibition and IV) representative excised tumors from the treatment groups with minimal V) histological damage in major organs. Tumor images correspond to different treatment groups: (a) Control, (b) Free drugs, (c) PCL blank NFM, (d) MNPs@NFM + AMF, (e) DOX@NFM + AMF, (f) CUR@NFM + AMF, (g) DOX/CUR@NFM + AMF, (h) MNPs/DOX/CUR@NFM without AMF, and (i) MNPs/DOX/CUR@NFM + AMF [238]. (B) AMF-responsive PCL–Fe_3_O_4_/DOX electrospun fibrous bandage for localized tumor therapy demonstrating the potential of electrospun PCL–Fe_3_O_4_/DOX fibrous bandages as localized magnetically activated platforms for treating drug-resistant cancer models [221].

Vo et al. developed pH-sensitive magnetic PCL fibers containing iron oxide and ammonium bicarbonate for doxorubicin delivery in postsurgical settings [74]. AMF-induced heating scale with iron-oxide nanoparticle content, indicating that the thermal performance depends on total implanted magnetic dose and construct volume rather than composition alone. Chen et al. designed a flexible PCL nanofiber mesh containing MNP for drug-resistant breast cancer (Fig. 4 A), using doxorubicin and curcumin to reverse multidrug resistance [238]. The mesh enabled sustained drug release for up to two months, while particles generated hyperthermia under AMF, producing a combined chemo-hyperthermic effect. This study addresses residual and refractory tumor cells while reducing repeated systemic administration, supporting the translational potential of MREF-based cancer therapy. Nevertheless, the contribution of each treatment component must be separated rigorously, because a multifunctional construct is not necessarily superior if equivalent tumor control can be achieved with a simpler drug-loaded fiber,

These studies establish the feasibility of magnetically activated local cancer therapy but do not yet resolve the key translational questions. Most experiments use small constructs, short follow-up periods and tumor models that do not reproduce human-scale perfusion or heat loss.

### 5.2 Tissue engineering and regenerative medicine

MREFs are most compelling in regenerative medicine when the magnetic phase provides a function that passive fibers cannot, particularly repeated non-invasive mechanical stimulation after implantation or *in situ* reorientation of injectable fibers [13,215]. Relevant targets include critical-size bone and osteochondral defects [36,212,213,216,239], peripheral nerve gaps [13,61,145,240], tendon repair and more prospectively cardiac repair. For bone, osteochondral, tendon [210,214] and cardiac [32,56] applications, scaffold placement will generally require surgical implantation, whereas nerve repair may use implanted conduits or injectable magnetically alignable fiber fragments [61,222]. The strongest rationale for MREFs in this setting is therefore magneto-mechanical rather than magnetothermal, because remote deformation or alignment can deliver repeated physical cues without exposing thermally sensitive regenerating tissues to hyperthermia [210,[213], [214], [215]]. Their translational value will depend on demonstrating that magnetic stimulation produces a reproducible biological benefit beyond an otherwise identical non-magnetic scaffold and remains effective across clinically relevant defect sizes [215].

**Fig. 5:**
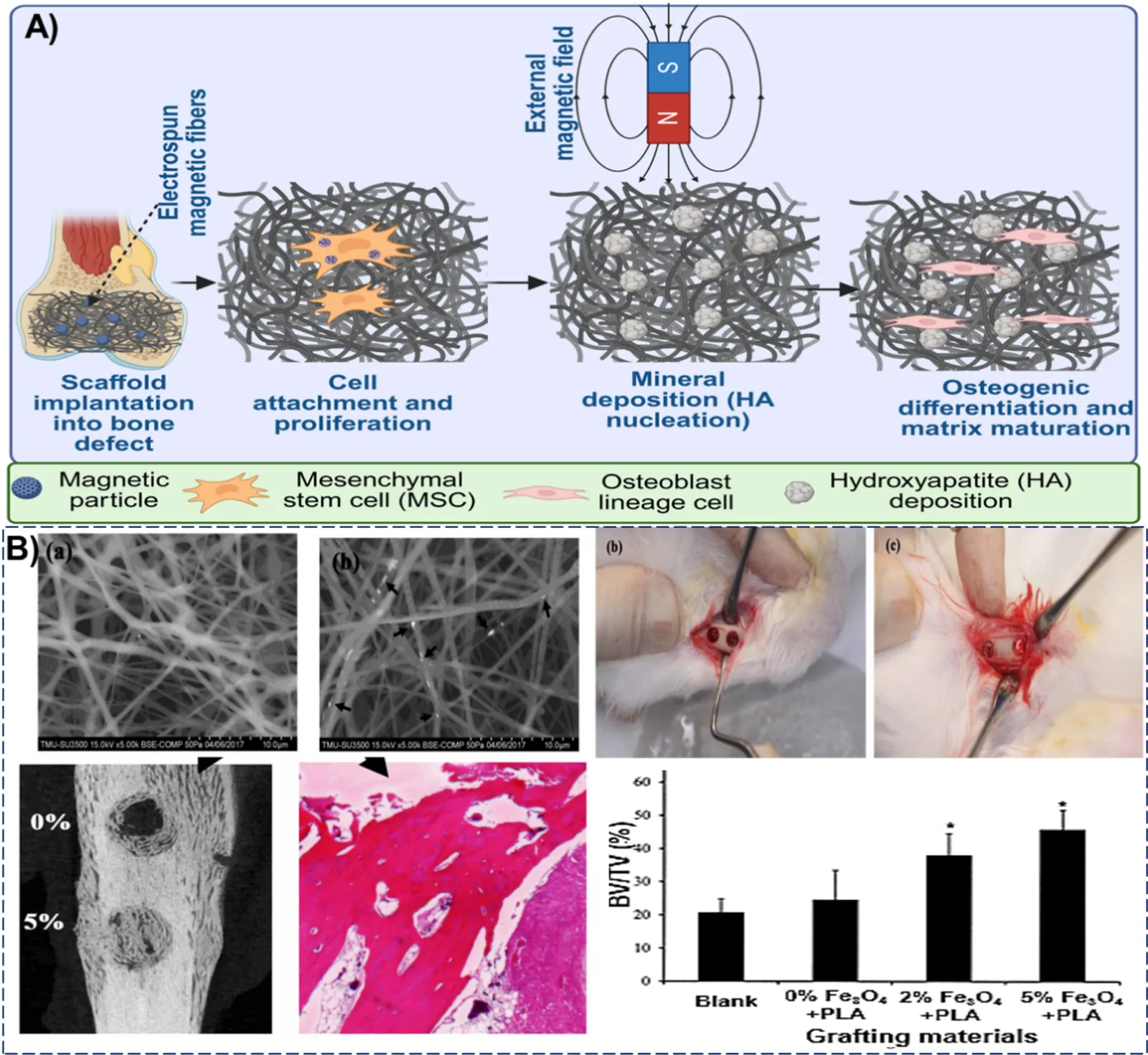
Magnetic electrospun fibers for tissue regeneration. (A) Schematic representation of magnetic electrospun fiber implantation into a bone defect. (B) SEM imaging confirms Fe_3_O_4_ particle incorporation within the PLLA nanofiber matrix, while surgical implantation, micro-CT/histological images, and quantification demonstrate an improved new bone formation in Fe_3_O_4_-containing PLLA grafts, especially at higher Fe_3_O_4_ loading [36].

Electrospun fibers provide topographical cues for cell attachment, migration, and alignment, while magnetic fillers introduce functions unavailable to passive scaffolds, including magneto-mechanical stimulation, scaffold positioning, magnetic guidance and imaging [241,242]. This combination is most useful when regeneration depends on both spatial organization and repeated physical stimulation. As illustrated in Fig. 5A, magnetic electrospun scaffolds can support bone regeneration by providing a fibrous matrix for cell attachment, mineral deposition, hydroxyapatite nucleation, osteogenic differentiation, and matrix maturation under magnetic-field-responsive conditions.

Bone regeneration is a plausible application because scaffold performance depends on mechanical competence, osteoconductive surface chemistry and mechanotransductive stimulation [241,243]. MREFs could integrate these functions, but magnetic effects must be distinguished from changes in porosity, stiffness and wettability caused by filler incorporation. Sadeghzadeh et al. developed PCL/collagen type I/Fe_3_O_4_ scaffold that improved mechanical strength, wettability, porosity, and biocompatibility relative to non-magnetic control and increased alkaline phosphatase activity, calcium deposition, and expression of COL1, RUNX2, osteocalcin, osteonectin and BMP2 in osteogenic cues-free media [65]. These findings support osteoinductive potential, although they do not isolate whether the response arose from magnetism or from filler-induced changes in scaffold structure and surface chemistry. Lai et al. (Fig. 5B) reported that Fe_3_O_4_/PLLA electrospun nanofibers implanted into rabbit tibial defects enhanced new bone formation, with 2% and 5% Fe_3_O_4_ producing 1.9- and 2.3-fold increases in bone volume, respectively, relative to untreated controls [36]. The greater effect at higher loading demonstrates a dose-dependent contribution of the magnetic phase, though only two loading levels were tested. However, the absence of a proportional relationship between loading and biological outcome suggests that increasing magnetic content alone is unlikely to provide a universal route to improved regeneration.

Magnetic actuation may be even more defensible in cartilage and osteochondral repair, where dynamic mechanical stimulation is biologically relevant. Zhang et al. incorporated magnetic nanoparticles into electrospun gelatin nanofibers to build a three-dimensional scaffold actuated by that moved a 0.5 Hz rotating magnetic field, generating mechanical stimulation through spatial confinement and fluid flow. The resulting scaffold motion and fluid displacement promoted MSC chondrogenesis, with citric-acid-coated particles increasing COL2A1 and ACAN expression. Following chondrogenic preconditioning, the cell-loaded magnetic constructs improved osteochondral repair *in vivo* [225]. The study demonstrates a useful design principle in which magnetic actuation supplies a mechanical cue rather than heat. Its interpretation nevertheless remains limited by the difficulty of separating effects of particle coating, scaffold movement and fluid shear.

**Fig. 6:**
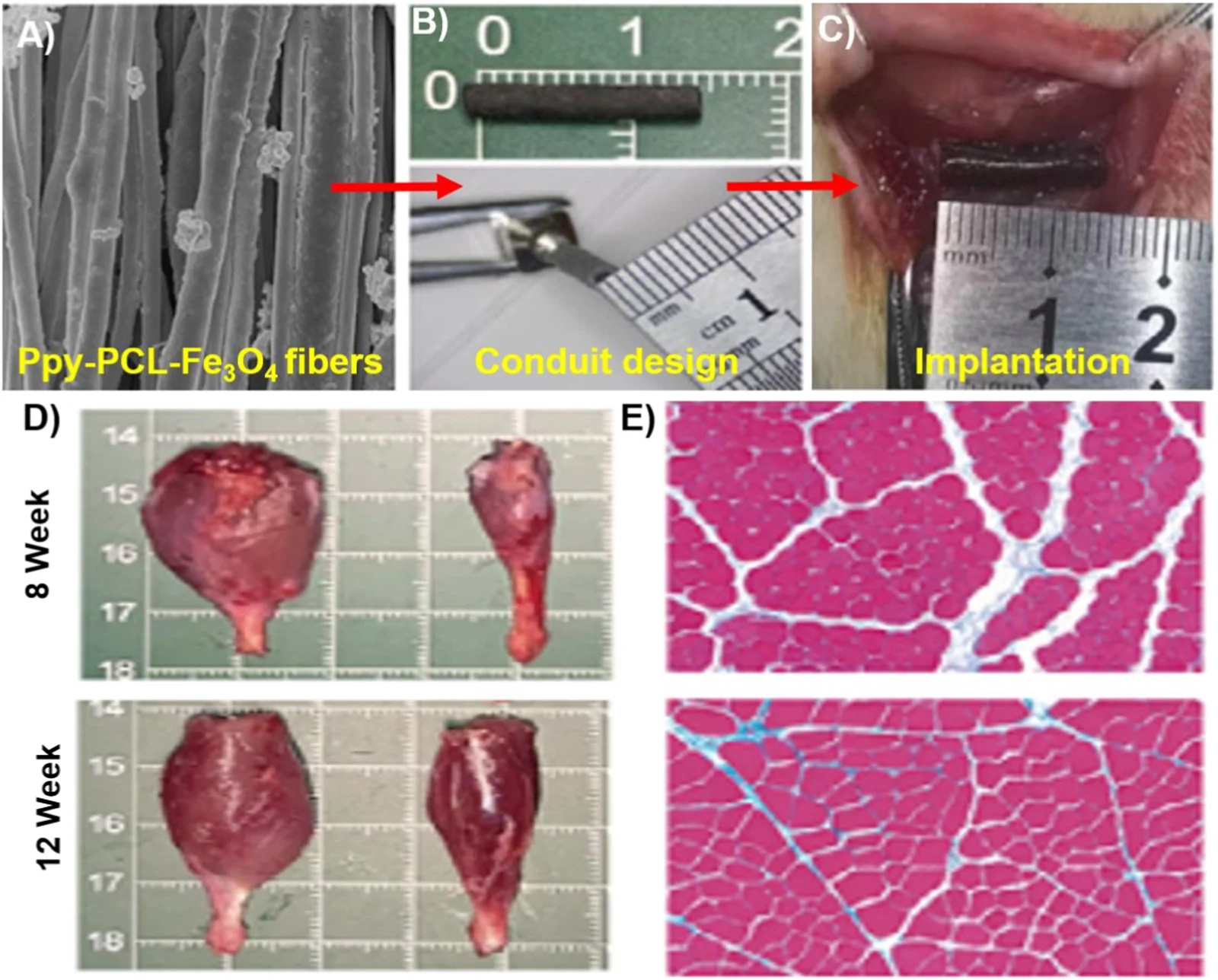
Ppy–PCL–Fe_3_O_4_ electrospun fibers for peripheral nerve regeneration**. (A–C)** SEM imaging shows the morphology of Ppy–PCL–Fe_3_O_4_ fibers, which were fabricated into a nerve-guidance conduit and surgically implanted at the nerve-defect site. **(D–E)** Gross muscle images and histological staining at 8 and 12 weeks demonstrate improved muscle preservation and tissue recovery after conduit implantation, indicating enhanced functional nerve regeneration [61].

Peripheral nerve regeneration is among the clearest areas where MREFs provide a direct regenerative advantage (Fig. 6A). Ppy–PCL–Fe_3_O_4_ electrospun fibers were fabricated into a nerve-guidance conduit and implanted at the nerve-defect site, where improved muscle preservation and histological recovery were observed after 8–12 weeks (Fig. 6) [61]. However, because these constructs combine conductive, magnetic and topographical cues, the specific contribution of magnetic actuation remains difficult to isolate. Studies from Gilbert and co-workers provide a more mechanistically focused basis for this application [13,144,145,222].

Johnson et al. developed injectable, magnetically orienting PLLA conduits containing oleic-acid-coated iron oxide nanoparticles [13]. The fibers could be delivered within collagen or fibrin hydrogels and aligned *in situ* using an external magnet. In dorsal root ganglion cultures, neurites extended approximately 1.4–3-fold farther along aligned magnetic fibers than through hydrogel alone. The principal value of this design is therefore not heating, but the ability to establish directional guidance after minimally invasive placement. Its translational relevance will depend on whether fiber alignment can be maintained in larger, mechanically dynamic defects after the external field is removed.

**Fig. 7:**
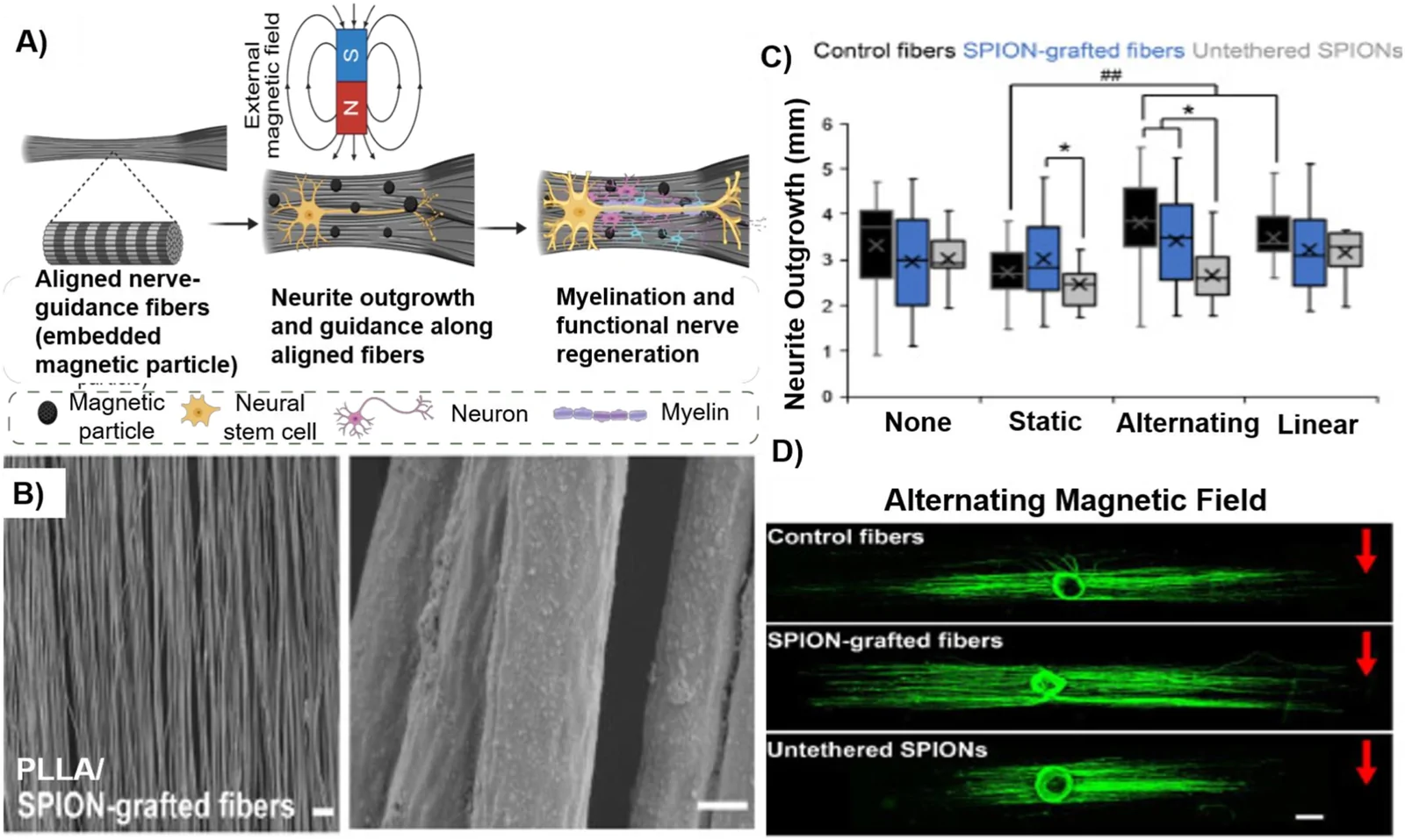
SPION-grafted aligned electrospun PLLA fibers for magnetically assisted peripheral nerve regeneration. (A) Schematic illustration of aligned nerve-guidance fibers containing embedded magnetic particles supporting neural stem-cell attachment, neurite extension, myelination, and functional nerve regeneration under an external magnetic field. (B–D) SEM images confirm the highly aligned morphology and surface grafting of SPIONs on electrospun fibers, demonstrating SPION-grafted fibers under alternating magnetic-field stimulation enhance directional neurite extension compared with control fibers or untethered SPIONs [222].

Funnell et al. compared aligned PLLA fibers, SPION-grafted fibers and untethered SPIONs under static and alternating magnetic fields (Fig. 7B–D) [222]. AMF stimulation increased neurite length by 40% on control fibers compared to static-field exposure, while SPION-grafted fibers under AMF produced a 30% increase in neurite length and a 62% increase in neurite area compared with fibers exposed to untethered SPIONs. The comparison with untethered SPIONs is particularly informative because it begins to separate the effect of fiber-bound magnetic actuation from the biological effect of iron oxide exposure alone. Nevertheless, a complete mechanistic analysis would also require matched measurements of local heating, scaffold displacement and field-only effects.

Cardiac tissue engineering represents a more exploratory application. Nazari et al. developed SPION–casein core–shell silk-fibroin nanofibers that supported embryonic cardiac cell attachment and enhanced expression of GATA4, cardiac troponin T, Nkx2.5, and α-myosin heavy chain compared to silk-fibroin fibers alone [223]. Kołodziejek et al. developed PU nanofibrous mats containing SPIONs that enabled structural and mechanical stimulation of cardiomyocytes under an external field [244]. These studies support the feasibility of magnetically active cardiac scaffolds, but they do not yet establish whether the magnetic phase provides a clinically meaningful advantage over conductive or mechanically conditioned non-magnetic fibers. Cardiac MREFs should therefore be considered promising but less mature than peripheral nerve applications.

### 5.3 Wound healing and antimicrobial dressings

**Fig. 8:**
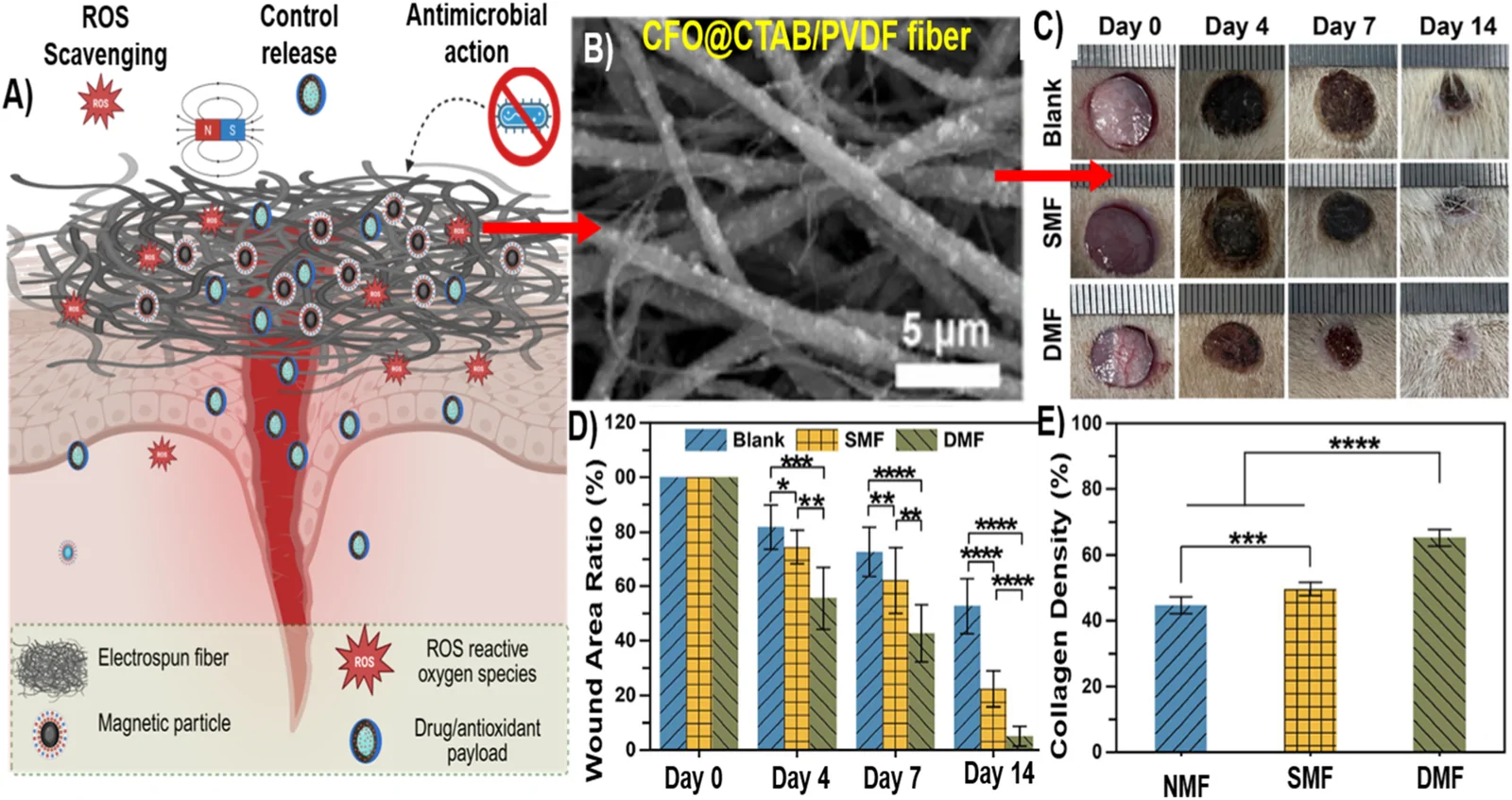
**Magnetic electrospun fibers for wound healing and antimicrobial therapy. (A)** Schematic illustration demonstrating magnetic electrospun fibers that promotes wound repair through ROS scavenging, sustained drug/antioxidant release, and antimicrobial activity. **(B–E)** SEM imaging confirms the fibrous morphology of CFO@CTAB/PVDF fiber mats, which provide an accelerated wound closure and enhanced collagen deposition and tissue regeneration, especially in the dual magnetic-field-treated group [143].

Wound care is one of the most translationally accessible applications of MREFs because superficial placement minimizes the field-penetration, coil-geometry and deep-tissue heating constraints associated with implanted systems (Fig. 8A). Relevant indications include diabetic foot ulcers, venous leg ulcers, pressure injuries and infected surgical wounds, typically treated with dressings spanning approximately 1–10 cm^2^. Potential advantages over passive dressing include on-demand release without repeated disturbance of the wound bed and localized magnetothermal stimulation. These benefits, however, must exceed those already achieved by advanced non-magnetic dressings that provide sustained release, moisture regulation and antimicrobial activity. Beyond these functions, electrospun dressings can also incorporate antioxidant components that scavenge excess reactive oxygen species (ROS), an established contributor to chronic inflammation and impaired closure in diabetic and other non-healing wounds; PCL/gelatin membranes loaded with the plant-derived antioxidant arbutin, for example, demonstrated effective radical scavenging alongside increased collagen deposition and accelerated wound closure *in vivo* [245]. The key evidence gap is therefore whether magnetic activation improves infection control or tissue repair sufficient to justify the additional material, device and safety burden [246,247].

Several reported systems demonstrate multifunctionality but provide limited evidence of magnetic actuation as the source of therapeutic benefit. Cai et al. showed that Fe_3_O_4_ improved the mechanical and antibacterial performance of chitosan/gelatin fibers against *Staphylococcus aureus* and *Escherichia coli*, but these effects were observed without an applied field [67]. Similarly, the PVA–cellulose nanofibril/CQD–Fe_3_O_4_–rosemary system reported by Rooholghodos et al. combined magnetic particles, carbon quantum dots and a phytochemical extract, making the specific contribution of Fe_3_O_4_ difficult to isolate [48]. Eghbalifam et al. developed silver–iron oxide composites for biofilm control, but antimicrobial activity is dominated by silver unless field-dependent contribution from iron oxide is demonstrated [142]. These studies therefore support magnetic particles as multifunctional additives, but not necessarily as evidence of magnetically controlled wound treatment.

**Fig. 9:**
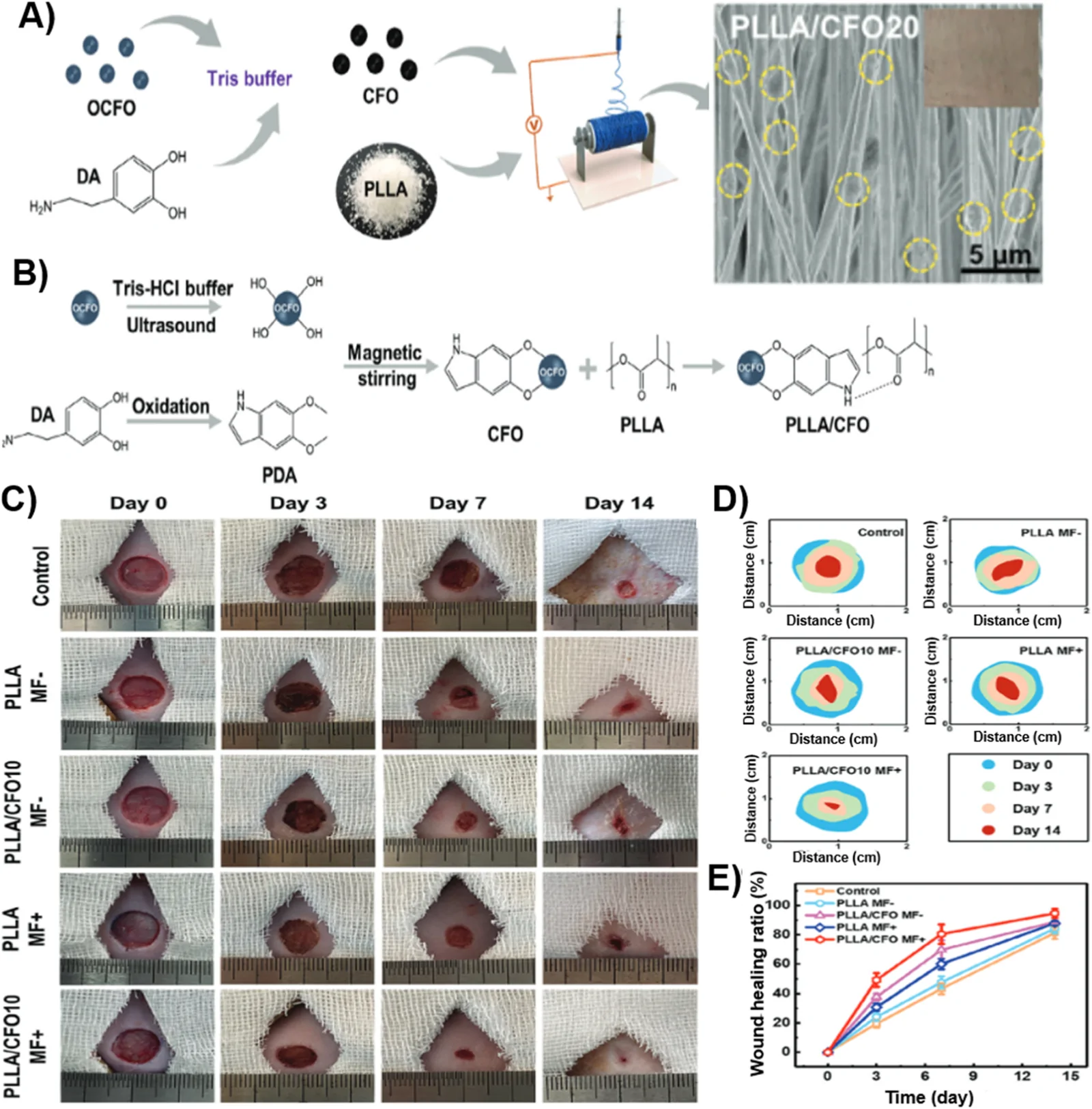
**PLLA/CFO magnetic electrospun fibers for magnetic-field-assisted wound healing. (A–E)** Schematic fabrication and surface modification of biodegradable PLLA/CoFe_2_O_4_ magnetoelectric nanofiber membrane prepared by electrospinning followed by dopamine-assisted CFO modification and used for *in vivo* evaluation in a full-thickness skin wound model under magnetic-field stimulation demonstrate accelerated wound closure and improve healing ratio after magneto–mechano–electric cascade stimulation [227].

Beyond passive antimicrobial activity, recent dressings introduce biophysical stimulation. Ke et al. combined CoFe_2_O_4_ with piezoelectric PVDF to develop CoFe_2_O_4_@CTAB/PVDF fibers which generate local electrical stimulation under a dynamic magnetic field, improving growth-factor expression and healing in infected diabetic wounds (Fig. 8B–E) [143]. Zhang et al. used PLLA/CoFe_2_O_4_ membranes to combine topographical guidance with magnetically induced piezoelectric stimulation (Fig. 9) [227]. These studies provide a stronger rationale for magnetic dressings because the external field actively modifies the therapeutic function rather than merely accompanying an antimicrobial material.

These interpretations nevertheless require caution. CoFe_2_O_4_ introduces cobalt-release concerns, although a removable topical dressing presents a lower exposure risk than a permanent implant. More importantly, the magnetic, mechanical and electrical contributions remain difficult to separate. Appropriate controls should include piezoelectric membranes without magnetic filler and magnetic membranes without a piezoelectric phase under identical field conditions. Without these comparisons, improved healing cannot be assigned specifically to the proposed magneto-mechano-electric cascade. Overall, wound applications remain promising because the dressing is accessible, retrievable and compatible with localized stimulation, but the field still lacks direct comparisons against state-of-the-art non-magnetic dressings.

### 5.4 Theranostics and biosensing

Theranostic MREFs combine magnetic actuation with imaging to confirm implant placement, guide field application and monitor persistence after implantation. Iron oxide is particularly attractive because the same phase can provide AMF heating and T_2_/T_2_*-weighted MRI contrast, limiting the need for separate imaging agents [13,25,31,149,248]. Reported systems include MRI-visible fibers for local chemotherapy, glioma implants and peripheral nerve repair. Their principal advantage is therefore not multifunctionality itself, but confirmation that the therapeutic scaffold has been accurately delivered and remains at the intended anatomical site.

Despite these advantages, MRI currently provides reliable qualitative localization but only limited quantification. Iron oxide produces a negative susceptibility contrast whose apparent signal void may extend beyond the implant because of blooming artifacts. The signal intensity depends on iron concentration, clustering, fiber orientation, field strength and imaging sequence making it unsuitable for accurately quantifying residual drug content, retained magnetic dose or scaffold degradation [249,250]. Signal voids are also non-specific and may be confounded by hemorrhaging, air or metallic artifacts commonly present in postoperative tissues. Current evidence therefore supports MRI for approximate localization, but not for accurate quantification of implant mass, drug content or degradation. Although progressive signal loss may indicate scaffold degradation or iron clearance, this relationship has not yet been validated against scaffold recovery or histological analysis [249,250].

Magnetic particle imaging (MPI) may overcome several of these limitations because it produces a positive, background-free signal that is directly proportional to iron content. In principle, MPI could permit quantitative assessment of retained magnetic dose, scaffold degradation and particle biodistribution, making it better suited than MRI for longitudinal monitoring. However, MPI application to MREFs remains unexplored and represents a clear research opportunity. Future studies should compare MRI and magnetic particle imaging, define detection limits and validate imaging signals against recovered scaffold mass and histological degradation [251,252].

**Fig. 10:**
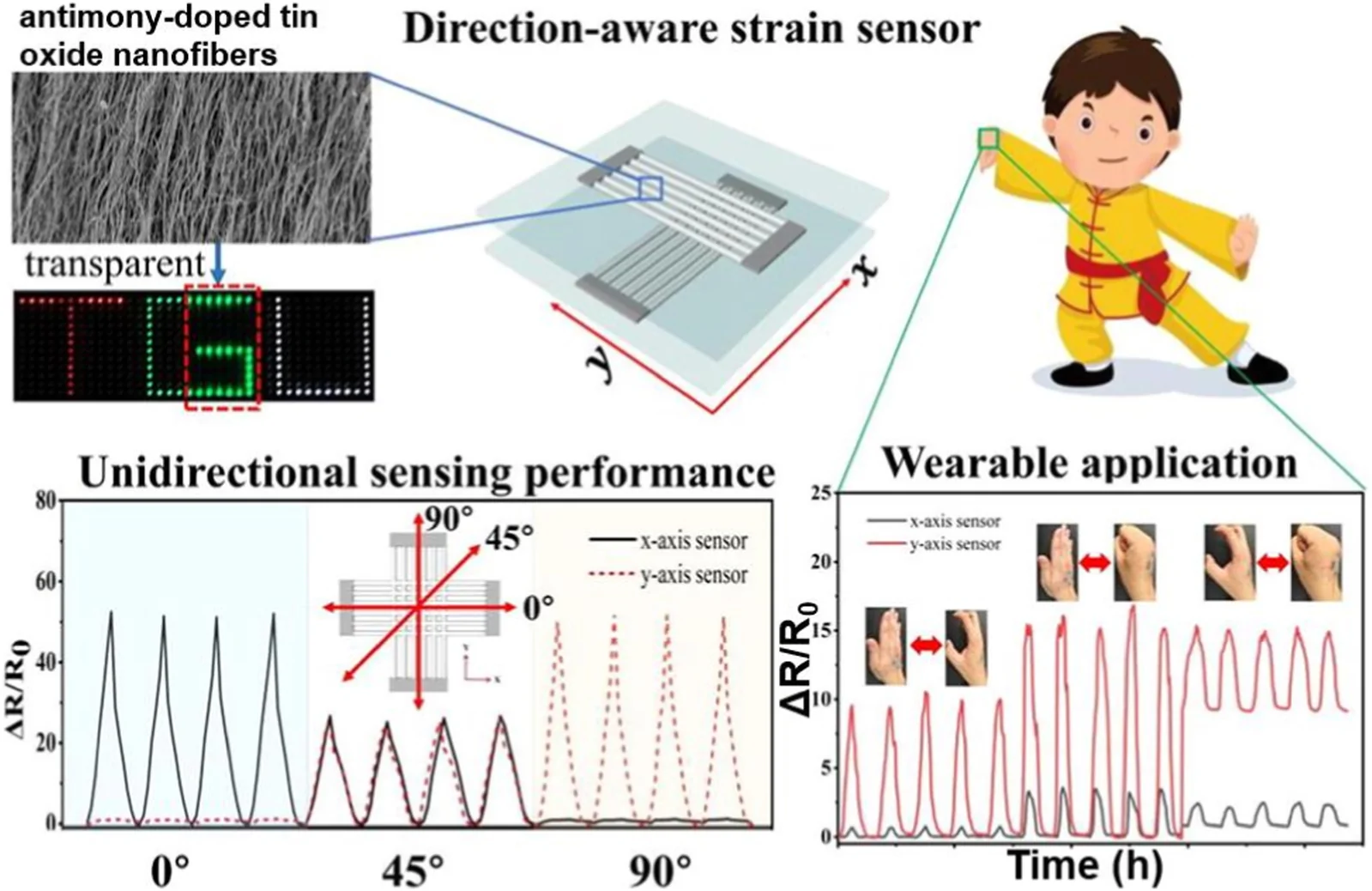
**Electrospun nanofiber-based direction-aware wearable strain sensor.** Electrospun antimony-doped tin oxide nanofiber films form a transparent anisotropic strain sensor capable of detecting strain direction and monitoring human motion [240].

Biosensing remains an emerging direction for MREFs. Bahrami et al. demonstrated an electrochemical sensor modified with electrospun magnetic nanofibers for morphine detection [253]. More broadly, electrospun biosensors have been developed for glucose, pressure, wearable physiological monitoring, and biomarker detection, because their porous structure increases analyte interaction, improves enzyme loading, and reduces diffusion resistance [254,255]. Yang et al. demonstrated a transparent, direction-sensitive strain sensor based on aligned electrospun fibers (Fig. 10) [240]. Although these systems are largely non-magnetic, they provide a foundation for future MREF biosensors in which magnetic particles could add remote manipulation, magnetically enhanced mass transport, or magnetically assisted signal modulation [239]. Such integration could ultimately enable closed-loop therapeutic scaffolds capable of sensing local biological changes and responding through magnetic actuation or on-demand drug release [256].

## 6 Biosafety, biodistribution and translational barriers

MREFs are intended for implantation or local placement, so translational assessment must extend beyond magnetic and drug-release performance to the biological fate of both the magnetic phase and the polymer matrix. Immobilizing within fibers may reduce immediate systemic exposure, but polymer degradation ultimately releases the particles and their surface coatings. Iron oxide remains the most defensible implantable filler because of its clinical precedent and physiological iron-handling pathway, yet its safety still depends on dose, coating, aggregation and clearance kinetics [15,257,258]. Surface-decorated particles require scrutiny because detachment under flow or mechanical loading may eliminate the localization advantage of the fibrous matrix [122,125,189,191].

The major evidentiary gap is the mismatch between degradation and biological follow-up. PCL- and PLGA-based constructs may release particles over months or years depending on composition and molecular weight [33,34,38,79]. Most MREF studies however evaluate biological responses only over days or weeks, leaving the long-term release and fate of magnetic fillers unresolved. Polymer degradation can also influence particle behavior. PLGA hydrolysis generates acidic products and may create a locally acidic microenvironment [87,88], while acidic conditions can accelerate PLGA erosion and iron oxide dissolution in PLGA–iron oxide composite systems [259,260]. Accordingly, polymer degradation, nanoparticle release, local iron accumulation and organ-level biodistribution should be evaluated as coupled processes rather than independent endpoints [257,258]. The greater magnetic performance of cobalt-, nickel- or manganese-containing ferrites does not remove the additional concern of metal-ion release and cumulative exposure [[104], [105], [106],[108], [109], [110],^118^]. Iron oxide should therefore remain the default implantable filler unless an alternative material provides an indispensable function.

Human-scale magnetic-field delivery is a second major barrier. AMF conditions that effectively heat a milligram-scale scaffold in a mouse cannot be transferred directly to a human-sized treatment volume. Increasing coil dimensions raises power and cooling requirements, reduces the ease of field confinement and increases the risk of non-specific eddy-current heating in healthy tissue [18,19,99,121]. Recent analyses further show that AMF tolerability depends on field amplitude, frequency, exposed anatomy and applicator geometry rather than on a universal H·f threshold [19]. Higher-SAR fillers may reduce the field required to achieve a target temperature, but they cannot eliminate the engineering and thermal constraints imposed by large treatment volumes. Magnetothermal MREFs are therefore most credible for superficial, surgically accessible or anatomically bounded sites.

Clinical relevance also depends on dose and treatment geometry. Heating is governed by the amount and distribution of magnetic filler and by tissue perfusion rather than by nanoparticle SAR alone [17,29,121,139]. Because SAR is defined per unit mass of magnetic material, the temperature rise achieved in a small laboratory sample does not scale directly to a perfused clinical target, where heat loss and field constraints dominate [261]. Similarly, MREF spatial precision is determined primarily by physical scaffold placement, whereas the magnetic field mainly provides temporal control of heating, mechanical actuation or release [2,3,13]. Future studies should therefore define the intended anatomical site, delivery route, total implant mass, elemental metal dose, field-exposed volume and achievable treatment region. Without this quantitative framework, claims of safe, precise and clinically scalable MREF therapy remain premature.

These barriers do not affect all delivery platforms equally. Table 9 compares with simpler or more established alternatives and identifies the circumstances in which remote magnetic actuation and fibrous architecture provide sufficient benefit to justify the added biological, manufacturing and regulatory burden.

**Table 9:** Comparative positioning of MREFs against competing platforms.

Platform	Spatial control	Post-placement temporal control	Structural or mechanical function	Delivery burden	Depth limitation	Advantages over MREFs	Refs
Conventional electrospun fiber	Defined by implant placement	Release fixed mainly during fabrication	Anisotropic topographical guidance without remote actuation	Surgical or topical	Determined by implant accessibility	Simpler manufacture and regulation; no magnetic-filler burden	[57,141,262,263]
Injectable hydrogel	Conforms to irregular defects	Possible with stimuli-responsive formulations	Bulk support but limited directional topography	Minimally invasive injection	Determined by needle or catheter access	Better defect filling and less invasive placement	[64,264,265]
Free magnetic nanoparticles	Limited by biodistribution and magnetic-gradient reach	Good AMF responsiveness	No persistent fibrous scaffold or contact guidance	Systemic or local injection	Magnetic targeting becomes less effective with depth	Avoids scaffold implantation and can be administered systemically	[2,15,121]
Microneedle array	Precise but superficial	Formulation- or device-dependent	Limited structural support	Minimally invasive	Restricted mainly to skin and superficial tissues	Better suited to transdermal delivery without surgical implantation	[[266], [267], [268]]
Implantable pump or electronic depot	Precise at the implantation site	Programmable and potentially closed-loop	No ECM-like fibrous guidance	Requires implantation and device hardware	Can operate at surgically accessible deep sites	Superior dose accuracy and programmability	[[269], [270], [271], [272]]
Magnetic soft robot or microrobot	Active navigation beyond the initial delivery site	Remote locomotion and on-demand payload release	Locomotion, deformation and local mechanical interaction	Injection or catheter-based delivery	Limited by field gradients, flow and anatomical geometry	Can navigate beyond the initial delivery site	[273,274]
MREF	Defined primarily by scaffold placement	Remote, repeatable and wireless	Combines fibrous topography with magnetic heating or mechanical actuation	Surgical, injectable-fragment or topical placement	Actuation depends on field geometry; MRI tracking is not similarly depth-limited	Integrates local delivery, anisotropic guidance and wireless actuation without batteries or leads	[13,61,139,145,275]

Comparisons represent a qualitative synthesis of representative studies rather than direct head-to-head testing. Performance depends on formulation, anatomical site, delivery route and actuation conditions.

The conclusion we draw is narrow but defensible. MREFs are most appropriate when the target is surgically or topically accessible, fibrous anisotropy provides an independent therapeutic benefit, repeated wireless stimulation is required, and implanted electronics are undesirable. These conditions are most clearly met in superficial or resection-cavity oncology, chronic wound care, and peripheral nerve or tendon repair. By contrast, deep, disseminated, or surgically inaccessible diseases remain poor candidates because field delivery, implant placement, and dose scaling limit the practical benefit of magnetic actuation.

## 7 Conclusions and future perspectives

Magnetic-responsive electrospun fibers represent a versatile biomaterials platform that integrates extracellular-matrix-like fibrous architecture with remote magnetic functionality. Within a single material system, MREFs combine scaffold topography, magnetic nanoparticles, controlled drug release, magnetothermal heating, magneto-mechanical stimulation, magnetic guidance, and imaging capability, demonstrating a distinctive capability in biomedical applications where localized placement and external activation are required. Rather than functioning as simple drug-loaded matrices, MREFs operate as responsive therapeutic scaffolds whose performance depends on the coordinated design of polymer chemistry, surface immobilization, fiber architecture and magnetic field parameters. Despite strong preclinical progress across multiple tissue targets, the principal barriers to clinical translation remain manufacturing scalability, batch-to-batch reproducibility, long-term magnetic-particle biodistribution, regulatory classification, clinically practical AMF delivery, and reliable *in vitro-in vivo* correlation.

**Fig. 11:**
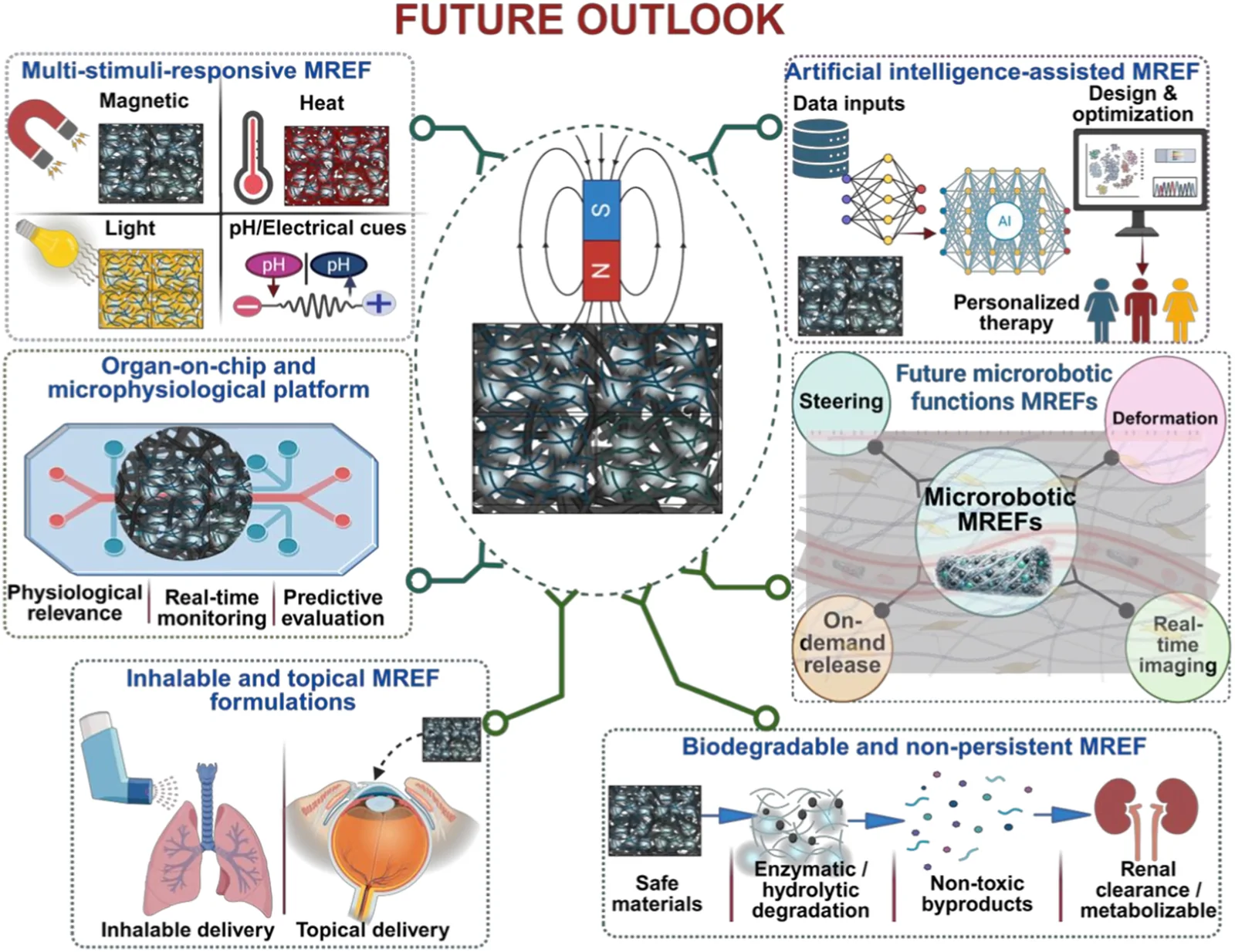
Future prospects of magnetic-responsive electrospun fibers (MREFs) in biomedical applications. The schematic summarizes emerging directions for next-generation MREF platforms.

Future progress will depend on the critical investment in translational infrastructure capable of converting laboratory-scale systems into regulated therapeutic products. The research and development priorities are likely to define the next phase of MREF development (Fig. 11).

**Multi-stimuli-responsive** MREF systems integrate magnetic responsiveness with pH-, thermo-, enzyme-, or light-sensitive release mechanisms, represent a next-generation precision pharmacotherapy platform. Thermoresponsive Fe_3_O_4_/PNIPAAm-based magnetic nanofibers have already been investigated for AMF-responsive drug delivery, while broader magnetic-stimuli-responsive nanosystems show how magnetic hyperthermia can be combined with stimuli-dependent drug release. pH- and magneto-responsive Fe_3_O_4_-containing polymer systems further support the concept of dual-responsive release [140,[276], [277], [278]].

**Artificial intelligence-assisted MREF** development addresses the multidimensional optimization challenges created by interacting variables such as nanoparticle size, surface chemistry, polymer concentration, solvent system, electrospinning voltage, flow rate, collector geometry, AMF parameters, and biological testing conditions which are inefficient to explore through traditional one-variable-at-a-time optimization. Recent studies have applied machine-learning models to predict electrospun fiber morphology and process–property relationships, while newer inverse-design and Bayesian-optimization frameworks show how data-driven methods can accelerate electrospun nanofiber development. The AI-guided electrospinning combined with automated formulation screening will support a practical strategy for optimizing MREF fiber diameter, nanoparticle loading, magnetization, release kinetics, and biological response [[279], [280], [281]].

**Organ-on-chip and microphysiological** platforms are promising tools for improving the predictive value of preclinical MREF testing. Static 2D cultures poorly reproduce tissue architecture, perfusion, mechanical cues, immune interactions, or drug-transport barriers that determine *in vivo* scaffold behavior. Tumor-on-chip systems have already been used to evaluate magnetic hyperthermia in glioblastoma models, where AMF exposure of iron-oxide-nanoparticle-loaded chips produced complete tumor-cell death within 30 min [282]. More broadly, thermo-regulatable magnetic microfluidic devices and organ-on-chip platforms provide a foundation for testing MREF-mediated hyperthermia, triggered release, and magneto-mechanical stimulation under more physiologically relevant conditions, bridging a gap between cell biology and *in vivo* animal models which can reduce animal burden and can be operated at industrial throughput for formulation screening [283].

**Inhalable and topical MREF** formulations may expand the field beyond surgically implanted scaffolds. For pulmonary delivery, aerodynamic particle size is a major determinant of deposition, and particles in the approximate 1–5 μm range are commonly discussed as favorable for deep lung delivery [284]. Magnetic aerosol studies further show that inhaled magnetic carriers can be focused or retained using external magnetic fields, although clinically relevant field gradients and tissue-depth limitations remain major challenges [285]. Topical and transdermal magnetic systems are also emerging, with recent work showing that magnetic components can support magnetically controlled transdermal delivery and AMF-induced heating [286,287].

**Biodegradable and non-persistent MREF** architecture will be important for addressing long-term biodistribution and safety concerns. *In vivo* studies of magnetic iron oxide nanoparticles identify biodegradation, systemic clearance, and iron metabolism as central translational challenges for magnetic biomaterials [257,258]. Ferritin-based and magnetoferritin systems offer a promising bioinspired alternative, as ferritin is a natural iron-storage protein capable of forming superparamagnetic iron oxide core within protein cage [114,288]. Magnetoferritin has also been evaluated for magnetic hyperthermia under AMF exposure, though its direct incorporation into MREF scaffolds remains a future research direction rather than an established translational solution [115,289].

**Magnetically actuated microrobotic fibers** represent an emerging extension of MREF from fixed implants to mobile therapeutic carriers. Injectable magnetic electrospun fiber fragments have demonstrated remote magnetic orientation after implantation while preserving ECM-like topographical guidance for cell alignment, suggesting the feasibility of combining fibrous architecture with magnetic control [13]. Recent advances in magnetic electrospun scaffolds and magnetically responsive biomaterials indicate that remote steering, deformation and on-demand release may eventually be integrated into fiber-based systems [5,21,25,121]. However, these capabilities remain largely conceptual for MREFs. Navigation in complex tissue, real-time imaging, payload retention, retrieval and biodegradation must be demonstrated under physiological flow and anatomical constraints before claims of deep-tissue delivery are credible. Direct comparison with hydrogels, magnetic nanoparticle suspensions, microneedles, implantable depots and soft microrobots will be essential to define whether mobile fibrous magnetic carriers provide meaningful advantages over existing platforms.

Future development should prioritize fabrication, physiologically relevant testing models, biodegradable magnetic components, and targeted safety validation. The most realistic early clinical opportunities lie in locoregional applications where MREFs offer a clear benefit over systemic therapy. With coordinated progress in materials engineering, pharmacology, magnetic-field delivery, and regulatory design, MREFs have the potential to move from advanced preclinical systems toward clinically useful, remotely controllable therapeutic technologies.

## Ethics approval and consent to participate

This review article does not involve new experiments using human participants, human tissue, animals or clinical data. Ethics approval and consent to participate are not required.

## Declaration of generative AI and AI-assisted technologies in the manuscript preparation process

During the preparation of this work, the author(s) used ChatGPT for the correction of spelling, grammatical errors, and language correction. The author(s) reviewed and edited the output as needed and take full responsibility for the content of the published article. No artificial intelligence participated in generating original scientific content presented in this work.

## Funding

No grant funding was used for the generation of the manuscript.

## CRediT authorship contribution statement

**Ashish Trital:** Conceptualization, Investigation, Supervision, Writing – original draft, Writing – review & editing. **Burhan Ates:** Conceptualization, Writing – review & editing. **Ryan J Gilbert:** Funding acquisition, Investigation, Project administration, Resources, Supervision, Writing – review & editing.

## Declaration of competing interest

The authors declare that they have no competing financial interests or personal relationships that could have influenced the preparation or the work reported in this review article.

## Figures and Tables

**Table:** 

Abbreviation	Full Term
AMF	Alternating magnetic field
CAP	Cellulose acetate phthalate
CE	Conformité Européenne (European Conformity) marking
CFO	Cobalt ferrite (CoFe_2_O_4_)
CNT	Carbon nanotube
CoFe_2_O_4_	Cobalt ferrite
CT	Computed tomography
CTAB	Cetyltrimethylammonium bromide
CUR	Curcumin
DOX	Doxorubicin
ECM	Extracellular matrix
EMA	European Medicines Agency
EPR	Enhanced permeability and retention (effect)
FITC	Fluorescein isothiocyanate
GO	Graphene oxide
IONP	Iron oxide nanoparticle
LCST	Lower critical solution temperature
MNP	Magnetic nanoparticle
MOF	Metal–organic framework
MPI	Magnetic particle imaging
MREFs	Magnetically responsive electrospun fibers
MRI	Magnetic resonance imaging
MSC	Mesenchymal stem cell
NFM	Nanofiber mat
PAN	Polyacrylonitrile
PANI	Polyaniline
PBS	Poly(butylene succinate)
PCL	Polycaprolactone
PDA	Polydopamine
PEDOT:PSS	Poly(3,4-ethylenedioxythiophene):poly(styrenesulfonate)
PEO	Poly(ethylene oxide)
PGA	Poly(glycolic acid)
PGS	Poly(glycerol sebacate)
PHBV	Poly(3-hydroxybutyrate-co-3-hydroxyvalerate)
PLGA	Poly(lactic-co-glycolic acid)
PLLA/PLA	Poly(L-lactic acid)/poly-L-lactide
PMMA	Poly(methyl methacrylate)
PNIPAAm	Poly(N-isopropylacrylamide)
PPy	Polypyrrole
PU	Polyurethane
PVA	Poly(vinyl alcohol)
PVDF	Poly(vinylidene fluoride)
SLP	Specific loss power
SPION	Superparamagnetic iron oxide nanoparticle
